# Hepatic Tumor Cell Morphology Plasticity under Physical Constraints in 3D Cultures Driven by YAP–mTOR Axis

**DOI:** 10.3390/ph13120430

**Published:** 2020-11-28

**Authors:** Adam Frtús, Barbora Smolková, Mariia Uzhytchak, Mariia Lunova, Milan Jirsa, Martin Hof, Piotr Jurkiewicz, Vladimir I. Lozinsky, Lucie Wolfová, Yuriy Petrenko, Šárka Kubinová, Alexandr Dejneka, Oleg Lunov

**Affiliations:** 1 Department of Optical and Biophysical Systems, Institute of Physics of the Czech Academy of Sciences, 18221 Prague, Czech Republic; frtus@fzu.cz (A.F.); smolkova@fzu.cz (B.S.); uzhytchak@fzu.cz (M.U.); mariialunova@googlemail.com (M.L.); sarka.kubinova@iem.cas.cz (Š.K.); 2 Institute for Clinical & Experimental Medicine (IKEM), 14021 Prague, Czech Republic; miji@ikem.cz; 3 J. Heyrovský Institute of Physical Chemistry of the Czech Academy of Sciences, 18223 Prague, Czech Republic; martin.hof@jh-inst.cas.cz (M.H.); piotr.jurkiewicz@jh-inst.cas.cz (P.J.); 4 A.N. Nesmeyanov Institute of Organoelement Compounds, Russian Academy of Sciences, Vavilov Street, 28, 119991 Moscow, Russia; loz@ineos.ac.ru; 5 Department of Biomaterials and Biophysical Methods, Institute of Experimental Medicine of the Czech Academy of Sciences, 14220 Prague, Czech Republic; lucie.wolfova@iem.cas.cz (L.W.); yuriy.petrenko@iem.cas.cz (Y.P.); 6 Department of Tissue Engineering, Contipro a.s., 56102 Dolni Dobrouc, Czech Republic

**Keywords:** mechanotransduction, YAP, mTOR, autophagy, 3D cultures, cytoskeleton, cell plasticity

## Abstract

Recent studies undoubtedly show that the mammalian target of rapamycin (mTOR) and the Hippo–Yes-associated protein 1 (YAP) pathways are important mediators of mechanical cues. The crosstalk between these pathways as well as de-regulation of their signaling has been implicated in multiple tumor types, including liver tumors. Additionally, physical cues from 3D microenvironments have been identified to alter gene expression and differentiation of different cell lineages. However, it remains incompletely understood how physical constraints originated in 3D cultures affect cell plasticity and what the key mediators are of such process. In this work, we use collagen scaffolds as a model of a soft 3D microenvironment to alter cellular size and study the mechanotransduction that regulates that process. We show that the YAP-mTOR axis is a downstream effector of 3D cellular culture-driven mechanotransduction. Indeed, we found that cell mechanics, dictated by the physical constraints of 3D collagen scaffolds, profoundly affect cellular proliferation in a YAP–mTOR-mediated manner. Functionally, the YAP–mTOR connection is key to mediate cell plasticity in hepatic tumor cell lines. These findings expand the role of YAP–mTOR-driven mechanotransduction to the control hepatic tumor cellular responses under physical constraints in 3D cultures. We suggest a tentative mechanism, which coordinates signaling rewiring with cytoplasmic restructuring during cell growth in 3D microenvironments.

## 1. Introduction

Despite technical advances and numerous experimental evidence, the concept that the mechanical cues and properties of a culture environment play a crucial role in modulating cell behaviors only recently became widely accepted [1]. Stiffness of the extracellular matrix, nanotopography and biomechanical forces originating in the cellular microenvironment have been identified as major regulators that control basic cell properties, including cell plasticity, motility and proliferation [2,3,4]. Of note, during tumorigenesis, mechanical features of the tumor microenvironment are changed dramatically, affecting cellular functions [1,5]. Specifically, liver stiffness was shown to be associated with the development of fibrosis and liver cancer [6,7]. Generally, changes in physical properties of the cellular microenvironment are observed in various liver pathologies [8]. It has been recognized that there is a connection between the stiffness of the liver and the risk of hepatocellular carcinoma (HCC) development in patients with hepatitis C [6]. Liver stiffness values around 5.5 kPa are considered normal, whereas liver stiffness in the range of 10–75 kPa is associated with different liver cancers [6,9,10,11]. Overall, stiffer extracellular matrix (ECM) has been shown to accelerate migration of hepatocellular carcinoma cells [12] and to promote proliferation and chemotherapeutic resistance in hepatocellular carcinoma cells [7]. In fact, patients with higher liver stiffness were shown to be at a significantly higher risk for HCC [6]. On the other hand, it was revealed that cancerous tissue exhibits mechanical heterogeneity, bearing a substantially stiffer invasive front in comparison to the core of the tumor [13,14]. Additionally, low ECM stiffness (1–5 kPa) was shown to promote cancer stemness [15]. A recent study revealed that a soft ECM enhances the cancer stem cell phenotype of HCC cells [16]. However, molecular foundations that mediate microenvironment stiffness-induced development of liver pathologies, specifically tumor formation, are still not fully understood. Moreover, the majority of current research is focused on signaling in HCC cells, which is biased by rather stiff ECM (i.e., stiffness about 10–30 kPa), whereas little attention is given to molecular mechanisms affected by soft ECM (i.e., stiffness lower than 1 kPa) [17,18,19].

It is becoming evident that Hippo–Yes-associated protein 1 (YAP) pathway activity is regulated by matrix stiffness, cell density and shape [20,21]. Upon activation, YAP1 protein migrates to the nucleus and modulates cell proliferation, tissue growth and differentiation [22]. It has been identified that YAP1 activity positively correlates with chemoresistance, tumor heterogeneity, mesothelial-to-mesenchymal transition, fibrosis, metastasis and frequency of self-renewing cancer stem cells in different types of tumors, including liver cancer [23,24,25,26]. Although it is not clear how YAP1 is deregulated in cancer [23], emerging evidence suggests that the YAP protein functions as an oncogene in tumorigenesis [27]. Therefore, it is very important to understand how YAP activity is altered in tumors. In fact, it has been shown that mammalian target of rapamycin (mTOR) positively regulates YAP activity [28]. On the other hand, YAP itself may activate mTOR signaling to drive cellular proliferation [29]. Additionally, ECM and integrin receptors have been shown to promote mTOR signaling activity and, in such a way, to regulate malignant transformation [30].

Those studies suggest that YAP and mTOR signaling might be interconnected in a complex cellular microenvironment. As a consequence of YAP mechanosensitivity, it is feasible that mTOR together with YAP may participate in mechanosensing during tumorigenesis. Importantly, critical questions need to be addressed regarding whether these signals and regulations are physiologically relevant and how they control cancer cell plasticity to drive proper proliferation in a complex 3D microenvironment. Of note, it has been demonstrated that there is a link between microenvironmental inputs and cellular plasticity of normal and tumor cells [31,32]. However, it is still unclear how cellular plasticity is connected mechanistically to a complex 3D microenvironment, and how the YAP–mTOR axis can be modulated spatiotemporally by extrinsic signals. To the best of our knowledge, to date, there are no reports that have directly demonstrated regulation of the YAP–mTOR axis by mechanical cues in a complex 3D microenvironment.

Here, we report the convergence of YAP and mTOR signals under mechanical constraint. We found that the YAP–mTOR axis controls liver tumor cell plasticity in 3D cellular culture-driven mechanotransduction. A three-dimensional cellular culture offers distinct competing mechanical cues, i.e., adhesion and pressure that regulate YAP and mTOR activity and subcellular localization. To a greater extent, our findings provide clues in YAP–mTOR-driven mechanotransduction regulation of the hepatic cellular responses under physical constraints in 3D cultures.

## 2. Results

### 2.1. Characterization of Collagen Scaffolds

**Figure 1:**
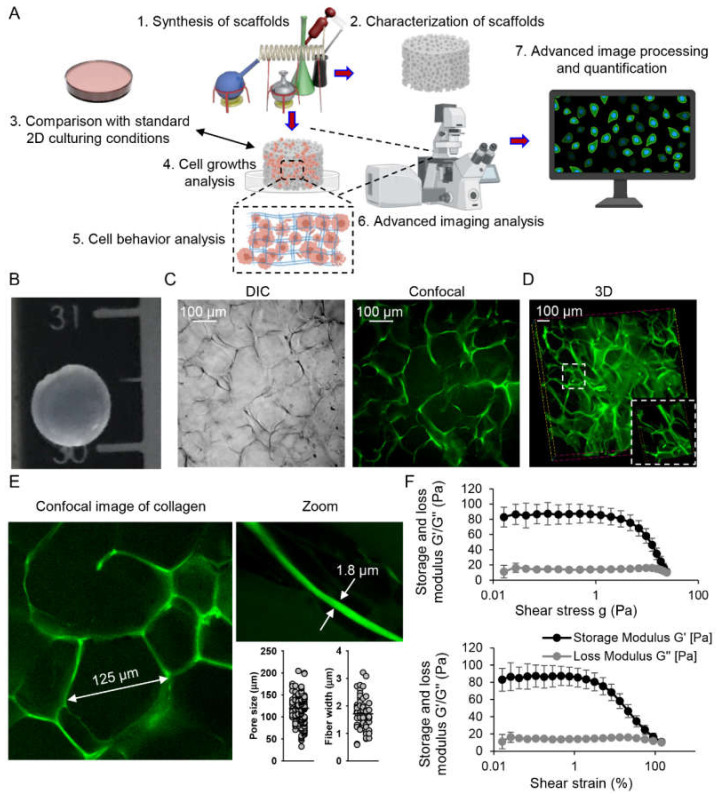
Synthesis and characterization of porous collagen scaffolds. (**A**) The schematic concept of the study. (**B**) A prepared 3D collagen scaffold representative image. (**C**) Representative images of a collagen scaffold captured by differential interference contrast (DIC) and confocal microscopy. (**D**) Three-dimensional reconstruction of a collagen scaffold morphology as captured by confocal microscopy. Three-dimensional reconstruction was done using ImageJ software (NIH). Insert shows zoomed region of the collagen scaffold. (**E**) High-resolution fluorescence confocal imaging of the collagen scaffold with subsequent quantitative assessments of pore size (*n* = 183) and fiber width (*n* = 57). Quantitative assessments were done using ImageJ software (NIH). (**F**) Viscoelastic properties of collagen scaffolds as determined by plate–plate crosshatched geometry in oscillatory strain sweep mode with frequency 1 Hz. Figure 1A was created using *BioRender.com*.

To study the effect of heterogeneous physical cues on cellular plasticity, we needed a model that would possess 3D architecture and allow the development of in vitro 3D biomimetic environments. Additionally, we wanted to compare cellular responses that occurred in 3D environments with conventional two-dimensional (2D) culturing (Figure 1A). In fact, recent research advances provide various options for 3D culturing, which supply cells with more realistic biochemical and biomechanical microenvironments [33,34,35].

Collagen is a major component of liver ECM and its reorganization is attributed to different liver pathologies, including cancer [36,37,38]. Specifically, deregulated enrichment of ECM with collagen I has been identified as a major ECM remodeling source during cancer progression [39,40]. Therefore, we selected 3D collagen scaffolds as a reliable model of 3D biomimetic cancer-related environments. To develop biomimetic scaffolds that recapitulate the liver tumor microenvironment, it is necessary to know the respective basic characteristics of the ECM in vivo. However, the literature is very scant in that aspect [41,42]. In fact, very little is known about collagen size characteristics in the liver. There are tentative estimations of collagen fiber diameter, thought to be in the range of 200–1000 nm [42,43,44]. Taking into account that such measurements are taken using electron microscopy, requiring sample drying and fixation, one would assume that in a liquid environment, collagen fibers can be >1 μm in diameter. The ECM of cirrhotic human livers was shown to bear pores of an approximate size of ~150 µm [42,45].

The collagen scaffolds were prepared as described previously [46]. Collagen scaffolds in the shape of a disc with a dimension of 6-mm diameter and 2-mm thickness were synthesized (Figure 1B–E). It is known that during chemical fixation and sample preparation for scanning electron microscopy evaluation, 3D scaffolds may dramatically change size and shape [47]. Thus, to evaluate morphology of prepared collagen scaffolds in a native liquid environment, we utilized ultrafast high-resolution fluorescence imaging with the novel IXplore SpinSR Olympus spinning disk confocal system [48,49].

Detailed confocal microscopy analysis revealed an average pore size of about 115 μm and a fiber width of ~2 μm of collagen scaffolds (Figure 1E). To assess the mechanical properties of the collagen scaffolds, the collagen samples were subjected to rheological analysis (Figure 1F). Indeed, collagen scaffolds had a storage modulus with average value G’ ~94 Pa. In fact, we selected those scaffolds for our cell experiments because they possess characteristics similar to liver ECM of pathological conditions. As we indicated above, collagen fiber diameter in the liver is estimated to be in the range of 200–1000 nm [42,43,44]. The pore size of cirrhotic liver ECM was assumed to be ~150 µm [42,45]. Moreover, low ECM stiffness (1–5 kPa) is associated with cancer stemness [15]. It is worth noting here that there are a number of commercially available 3D scaffolds. Matrigel and Alvetex^®^ are most commonly used, especially in liver cell cultures [50,51]. However, Matrigel possesses fibers in the range of 70 nm in diameter [52]. The average pore size of Matrigel is about 2 μm [53]. Alvetex^®^ represents a highly porous polystyrene scaffold designed for 3D cell cultures. The void dimensions of the scaffold are ~36–40 μm in diameter and interconnects are of ~12–14 μm in diameter [54]. Thus, our scaffolds represent a very good model to study liver cancer cell plasticity under mechanical constraints of native extracellular microenvironment.

Furthermore, we analyzed the diffusional properties of different molecules in collagen scaffolds using fluorescence correlation spectroscopy (FCS) (see Table 1 and Supplemental Figure S1). The diffusion time measured for the small water-soluble fluorescent probe Alexa 488 was slower in the region of collagen fibers than in the solution or inside scaffold pores; i.e., the fraction of the longer diffusion time, which was also observed inside of the pores, becomes larger and the longer diffusion time increases. We found that the strong adsorption of many fluorescent probes (including Alexa 488 and Atto 488) to collagen scaffolds is a limiting factor in diffusion measurements. Larger molecules, such as polysaccharides, can also adsorb to the scaffolds. Dextran 3000 diffuses considerably slower when in between collagen fibers. A small fraction of the dextran molecules is immobilized by collagen. Larger dextran (MW ≈ 10,000 Da) molecules behave similarly, although the immobilized fraction becomes larger and, therefore, the uncertainty of the measured values increases. Small unilamellar liposomes formed from synthetic palmitoyl-oleoyl-phosphatidylcholine lipid were able to penetrate into the scaffold pores to some extent. The typical diffusion times for the small vesicles observed within the pores disappeared between collagen fibers. Immobilization of lipids was much stronger than that of dextrans. Lipids also penetrated the collagen matrix less deeply. Partial formation of a supported phospholipid bilayer has been observed on the fibers forming the walls of scaffold pores (Supplemental Figure S1).

### 2.2. HepG2 and Alexander Cells Change Their Size and Shape during Growth in Collagen Scaffolds

The majority of studies on mechanotransduction are performed using only one cell line without direct comparison of the observed effects on closely related cell lineages [2,3,4]. It is worth noting that liver cell cultures represent an interesting cell model to mechanoresponses, because improving hepatic culturing conditions is a major challenge for drug discovery and regenerative medicine of liver pathologies [55,56,57]. Thus, in this study, we chose two hepatic cell lines (HepG2 and Alexander cells).

**Figure 2:**
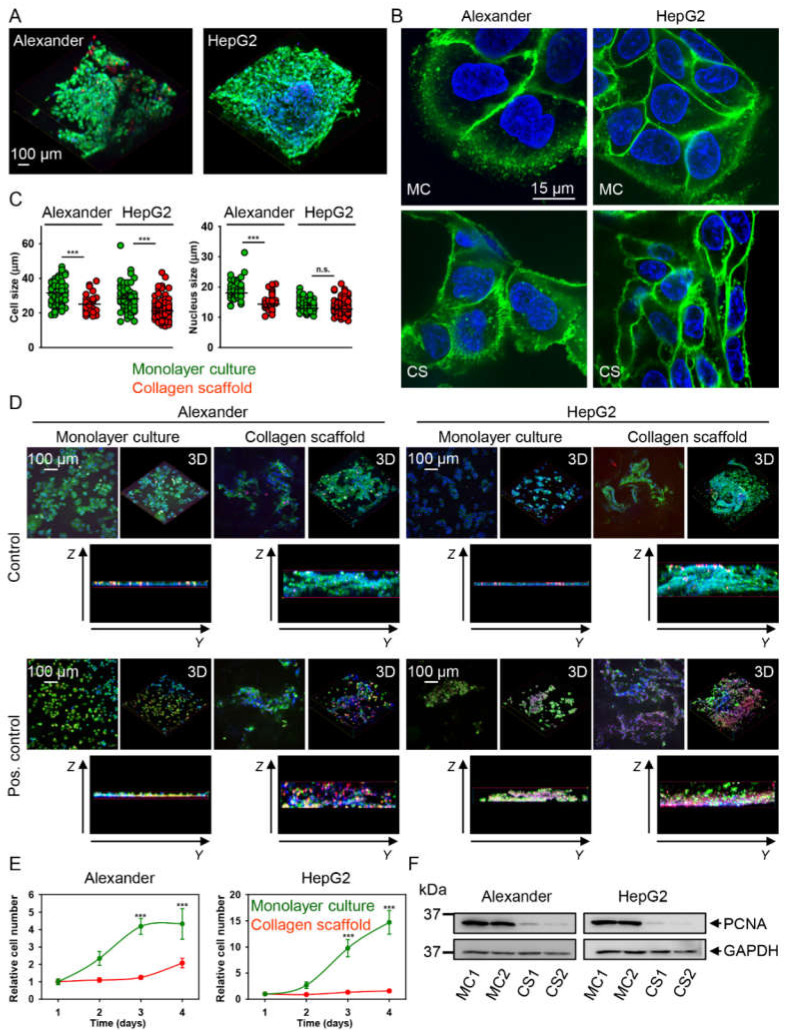
Hepatic cell size and plasticity under culturing in collagen scaffolds. (**A**) Viability assessment. Cells were loaded with calcein-AM (green) and ethidium homodimer (red); images were acquired by confocal microscopy. Hoechst 33342 (blue) dye was used to counterstain nuclei. Three-dimensional reconstruction was done using ImageJ software (NIH). (**B**) HepG2 and Alexander cells were grown either in standard monolayer culture (MC) or in collagen scaffolds (CS). Cell membranes were labeled with CellMask™ Green (green). Hoechst 33342 (blue) dye was used to counterstain nuclei. Labeled cells were then imaged by confocal microscopy and the images were processed using ImageJ software (NIH). (**C**) Image quantitative analysis of cell and nuclei sizes was performed in ImageJ software (NIH). Quantifications are presented as means of *n* = 27–86 cells, (***) *p* < 0.001 denotes significant differences. (**D**) HepG2 and Alexander cells were grown either in a standard monolayer culture or in collagen scaffolds. Cell were labeled with CellMask™ Green (green), as a membrane stain, and propidium iodide (red), as a dead cell stain. Hoechst 33342 (blue) dye was used to counterstain nuclei. Control cells were untreated. As a positive control, cells were treated with 20% ethanol for 60 min. Labeled cells were then imaged by confocal microscopy. ImageJ software (NIH) was used for image processing and 3D reconstruction. (**E**) Growth curves of HepG2 and Alexander cells either in standard monolayer culture or in collagen scaffolds, (***) *p* < 0.001 denotes significant differences. (**F**) Proliferating cell nuclear antigen (PCNA) expression was determined by immunoblotting; GAPDH—loading control. HepG2 and Alexander cells were grown either in a standard monolayer culture (MC) or in collagen scaffolds (CS).

First of all, we wanted to select a single time-point of cell growth in 3D collagen scaffolds, when sort-of-steady-state conditions are reached by cell proliferation and cells populate the majority of the scaffold. We cultured HepG2 and Alexander cells in collagen scaffolds for 1, 2, 3, 4 and 7 days (data not shown). Both cell lines sufficiently populated the collagen scaffolds at day 7 (Videos S1 and S2). Thus, we selected this time point for our detailed study on mechanotransduction in 3D conditions. Furthermore, we confirmed that such culturing for 7 days did not trigger a cytotoxic response in both HepG2 and Alexander cells (Figure 2A). Both cell lines showed dramatically decreased cell and nuclear sizes and changed shape after 7 days of culturing in collagen scaffolds (Figure 2B,C and Supplemental Figure S2).

In order to confirm that after a 7-day culture in 3D conditions, cells stay responsive to external stimuli, we checked the reaction of the cells to a toxic compound. Ethanol toxicosis represents a robust and fast toxicological and pharmacological model to verify the reactivity of hepatic cells [58,59,60]. In fact, both HepG2 and Alexander cells grown in collagen scaffolds showed responses to toxic ethanol dose comparable to the cells cultured in standard 2D monolayer conditions (Figure 2D and Supplemental Figure S3).

Furthermore, we evaluated the proliferative potential of cells grown in 3D collagen scaffolds versus standard 2D culturing. In both cell lines, culturing in collagen scaffolds slowed down cell proliferation (Figure 2E). Additionally, to confirm the impact of collagen scaffold culturing on proliferation, we assessed more specific cell proliferation markers, namely proliferating cell nuclear antigen (PCNA) and Ki-67 [61,62,63,64,65]. PCNA is a nuclear protein that is necessary for DNA synthesis during the G1/S phase of the cell cycle. It is well known that both quiescent and senescent cells have very low levels of PCNA [61,62,64,65].

PCNA protein levels were significantly lower in cells cultures in collagen scaffolds in comparison with the monolayer culture (Figure 2F), indicating that cell proliferation was decreased in collagen scaffolds. To validate these data, we analyzed another proliferation marker, i.e., Ki67. In fact, Ki-67 is expressed during the late G1, S, G2 and M phases of the cell cycle. However, quiescent or senescent cells lack Ki-67 expression [61,63]. Indeed, immunostaining analysis revealed that the majority of both Alexander and HepG2 cells grown in the monolayer culture were positive for Ki-67 (see Supplemental Figure S4). However, both cell lines showed a sharp decline in Ki-67-positive cells when grown in collagen scaffolds (see Supplemental Figure S4). Together, these data clearly imply that both cell lines transformed from a highly proliferative state in monolayer culture to low-proliferating cells in collagen scaffolds.

### 2.3. Cytoskeleton Remodeling and Modulation of YAP Signaling in Cells Grown in Collagen Scaffolds

It is not surprising that mechanical forces originating in 3D culture microenvironment alter fundamental cell properties, e.g., proliferation [2,3,4]. However, we hypothesized that cells in porous collagen scaffolds with a relatively large pore size and heterogeneous structure will exert distinct competing mechanical cues. Thus, we deeply analyzed cellular distribution in collagen scaffolds.

**Figure 3:**
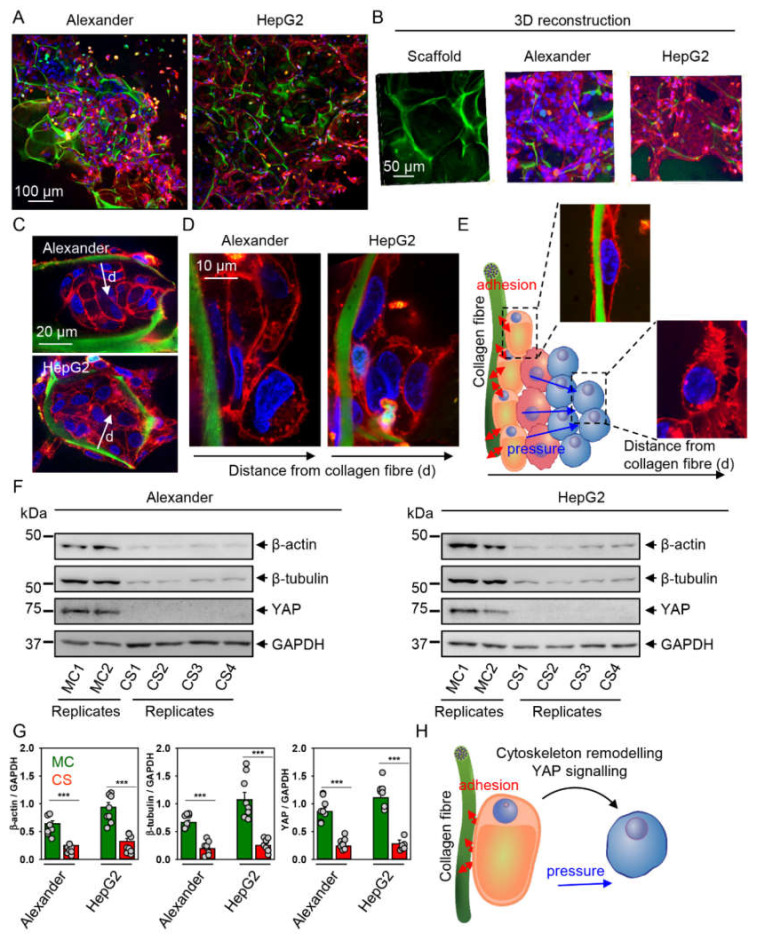
Cytoskeleton remodeling and downregulation of YAP signaling. (**A**) HepG2 and Alexander cells were grown in collagen scaffolds for 7 days. Cell membranes were labeled with CellMask™ Orange (red). Hoechst 33342 (blue) dye was used to counterstain nuclei. Collagen fibers were stained with Col-F (green). Labeled cells were then imaged by confocal microscopy and the images were processed using ImageJ software (NIH). (**B**) Three-dimensional reconstructions of either empty scaffolds or those populated with HepG2 or Alexander cells were done using ImageJ software (NIH). Labeling and imaging was done as in (**A**). (**C**) High-magnification imaging of cells inside collagen pores. Labeling and imaging was done as in (**A**). (**D**) High-resolution analysis of cell distribution in collagen scaffolds. Labeling and imaging was done as in (**A**). Arrow indicates the direction from collagen fiber. (**E**) The concept of distinct competing mechanical cues, which affect cell plasticity and functions in collagen scaffolds. (**F**) YAP, β-actin and β-tubulin expressions were determined by immunoblotting; GAPDH—loading control. HepG2 and Alexander cells were grown either in standard monolayer culture (MC) or in collagen scaffolds (CS). Representative blots out of three independent experiments are shown. Lysates from MC were loaded in duplicate; lysates from CS were loaded in quadruplicate. Uncropped blots and replicates are shown in the Supplementary Materials. (**G**) Graphs show densitometric quantification of blots. HepG2 and Alexander cells were grown either in standard monolayer culture (MC) or in collagen scaffolds (CS). The data are expressed as mean  ±  SEM, *n* = 5. (***) *p* < 0.001 denotes significant differences with respect to the monolayer culture. (**H**) Schematic illustration of distinct competing mechanical cues, i.e., adhesion and pressure, that regulate YAP activity.

Indeed, both HepG2 and Alexander cells populated the entire pore volume of collagen scaffolds within 7 days of culture (Figure 3A–C). For both Alexander and HepG2 cells, we observed consistent morphological changes in response to growth in collagen scaffolds. Cells close to the center of collagen pores were rounded, whereas stretched and flattened cells were observed on collagen fibers (Figure 3C,D). This observation led us to a suspicion that such a complex 3D microenvironment offers distinct competing mechanical cues—hypothetically, adhesion and pressure (Figure 3E).

Generally, it is known that cytoskeleton tension and reorganization conduct mechanical cues exerted on cells by the surrounding microenvironment [66,67,68,69]. Additionally, YAP pathway activity plays a central role in mediating the effects of mechanical stimuli on cellular behavior and is involved in important cell processes, such as development, proliferation, stemness, differentiation and tumorigenesis [20,21,26,70,71,72]. Indeed, physical cues have been shown to modulate cell proliferative capacity through mechanical regulation of YAP [21,70]. Interestingly, cytoskeletal dynamics, in particular actin, participates in the regulation of YAP signaling [70,73]. In turn, YAP regulates the turnover of F- and G-actin [74]. Therefore, we analyzed cytoskeletal dynamics and YAP expression in cells grown in collagen scaffolds. We found that both major cytoskeletal proteins (β-actin and β-tubulin) were dramatically downregulated in cells grown in collagen scaffolds (Figure 3F,G). These results suggest that such downregulation of the total levels of cytoskeletal proteins may contribute to the long-lasting alteration in the arrangement of the cytoskeleton. Additionally, levels of YAP protein were substantially lower in cells grown in the collagen scaffolds compared to the monolayer culture (Figure 3F,G). These results implied that YAP signaling and cytoskeletal remodeling could be involved in the observed lasting impact (Figure 2) of 3D culturing on cell size and morphology (Figure 3H).

**Figure 4:**
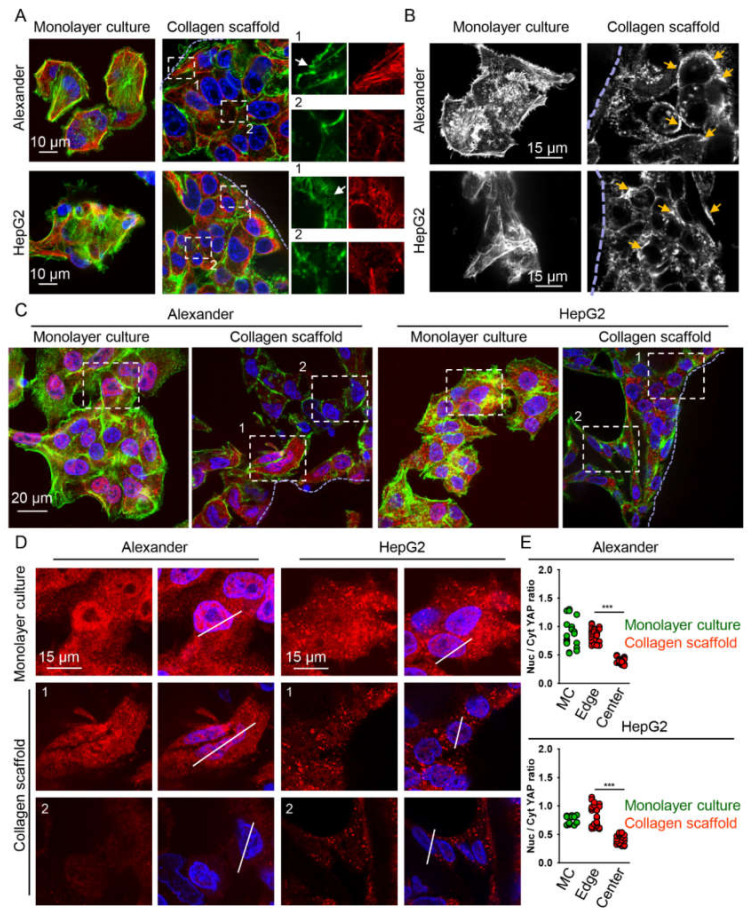
YAP cytosolic translocation under culturing in collagen scaffolds. (**A**) HepG2 and Alexander cells were grown either in standard monolayer culture or in collagen scaffolds. F-actin was labeled using ActinGreen™ 488 ReadyProbes™ reagent (green). Tubulin (red) was labeled using anti-β-tubulin antibody. Hoechst 33342 (blue) dye was used to counterstain nuclei. Labeled cells were then imaged by confocal microscopy and the images were processed using ImageJ software (NIH). Inserts represent zoom regions. The dashed lines highlight the collagen fiber–cells interface. Arrows indicate structures resembling filopodia. (**B**) Confocal fluorescence images (maximum intensity projection) of HepG2 and Alexander cells were grown either in standard monolayer culture or in collagen scaffolds for 7 days. F-actin was labeled using ActinGreen™ 488 ReadyProbes™ reagent. Labeled cells were then imaged by confocal microscopy and the images were processed using ImageJ software (NIH). The dashed lines highlight the collagen fiber–cells interface. Arrows indicate spots of increased F-actin tension. (**C**) Assessment of YAP sub-cellular localization. HepG2 and Alexander cells were grown either in standard monolayer culture or in collagen scaffolds. F-actin was labeled using ActinGreen™ 488 ReadyProbes™ reagent (green). YAP (red) was labeled using antibody. Hoechst 33342 (blue) dye was used to counterstain nuclei. Labeled cells were then imaged by confocal microscopy and the images were processed using ImageJ software (NIH). The dashed lines highlight the collagen fiber–cells interface. (**D**) Zoomed regions from corresponding confocal microscopic images shown in (**C**). (**E**) Assessment of nuclear/cytosolic YAP ratio of HepG2 and Alexander cells grown monolayer culture (green) or in collagen scaffolds (red). Sub-cellular localization of YAP under culturing in collagen scaffolds was analyzed at the edge and in the center of the pores of the scaffold. (***) *p* < 0.001 denotes significant differences, *n* = 18–24 cells per condition.

In fact, detailed high-resolution confocal imaging analysis revealed that both actin and tubulin cytoskeletal elements were dramatically remodeled in cells grown in collagen scaffolds (Figure 4A and Supplemental Figures S5 and S6). HepG2 and Alexander cells grown in the standard monolayer culture showed branched and multiple-directed F-actin stress fibers at the cell edge as well as traversing the whole cell body (Figure 4A,B). Cells grown in the collagen scaffolds demonstrated a loss of stress fibers that was accompanied by F-actin distribution, mostly at the cell periphery (Figure 4A,B). Close to collagen fibers, cells showed the presence of thin protrusions resembling filopodia, which were absent in cells located closer to the center of collagen pores (Figure 4A). Interestingly, cells located closer to the center of collagen pores demonstrated a number of bright spots of F-actin stress fibers located at cell–cell contacts (Figure 4B). Thus, we could tentatively conclude that cells close to collagen fibers were subjected to adhesion forces, whereas in the center of collagen pores, cells felt pressure from neighbors (Figure 3E). Consistent with this assumption, YAP localization confirmed that cells in the center and at the edge of collagen pores experienced distinct competing mechanical cues. Thus, cells at the edge of collagen pores showed marked nuclear localization of YAP (Figure 4C–E and Supplemental Figure S7). On the contrary, cells in the center of collagen pores demonstrated preferably cytosolic YAP localization (Figure 4C–E). Of note, the total amount of YAP protein was substantially lower in cells grown in collagen scaffolds compared to monolayer culture (Figure 4D), which was confirmed by immunoblot analysis (Figure 3F).

### 2.4. YAP–mTOR Signaling Interplay under Mechanical Stress in Collagen Scaffolds

**Figure 5:**
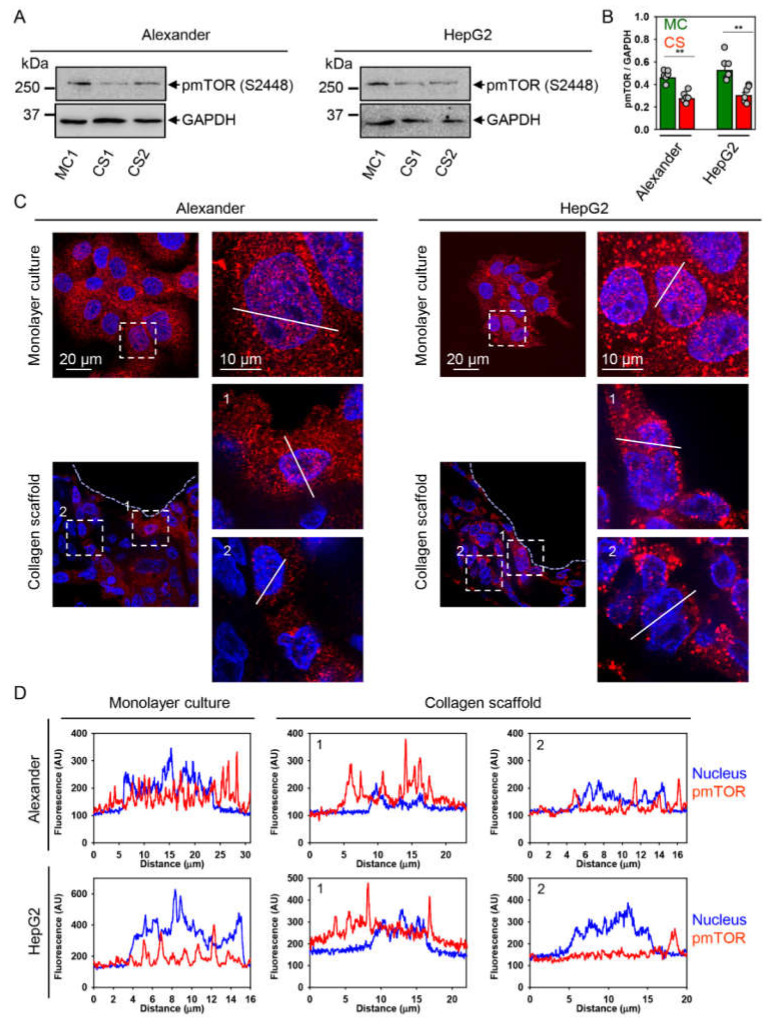
Mammalian target of rapamycin (mTOR) cytosolic translocation under culturing in collagen scaffolds. (**A**) Expression of phosphorylated mTOR (pmTOR) was analyzed by immunoblotting. GAPDH—loading control. HepG2 and Alexander cells were grown either in standard monolayer culture (MC) or in collagen scaffolds (CS). (**B**) Graphs show densitometric quantification of blots. HepG2 and Alexander cells were grown either in standard monolayer culture (MC) or in collagen scaffolds (CS). The data are expressed as mean  ±  SEM, *n* = 4. (**) *p* < 0.01 denotes significant differences with respect to monolayer culture. (**C**) Assessment of pmTOR sub-cellular localization. HepG2 and Alexander cells were grown either in standard monolayer culture or in collagen scaffolds, then pmTOR (red) was labeled using antibody. Hoechst 33342 (blue) dye was used to counterstain nuclei. Labeled cells were imaged by confocal microscopy and the images were processed using ImageJ software (NIH). Inserts represent zoom regions. The dashed lines highlight the collagen fiber–cells interface. (**D**) Sub-cellular localization of pmTOR under culturing in collagen scaffolds. Representative line scans of confocal microscopic images shown in (**C**).

It is worth noting here that mechanical stimuli may promote mTOR signaling [30,75,76]. Additionally, mTOR activity is associated with YAP nuclear-cytosol shuttling via mechanical-driven stimuli [28,29,30]. Therefore, we further evaluated mTOR activity and localization in cells grown in collagen scaffolds. Immunoblot analysis revealed that in cells grown in collagen scaffolds, the total level of active (phosphorylated) form of mTOR was significantly lower in comparison to monolayer culture (Figure 5A,B).

Sub-cellular localization analysis of the phosphorylated form of the mTOR (pmTOR) performed by confocal microscopy showed that pmTOR localization followed YAP sub-cellular distribution in cells grown in collagen scaffolds (Figure 5C,D). Specifically, cells located closer to the center of collagen pore displayed marked cytosolic pmTOR distribution, whereas in cells grown at the edge of collagen pores, pmTOR was observed in the nucleus as well (Figure 5C,D). Indeed, it is well established that classical mTOR signaling is mediated at the lysosomal surface [77,78,79]. However, emerging evidence suggests that phosphorylated mTOR can play an important role in the nucleus [80,81,82,83,84,85,86]. In fact, its role in the nucleus is not well determined [81,84]. It was found that increased mTOR nuclear localization might be related to poor prognosis of different types of cancer [83,87].

**Figure 6:**
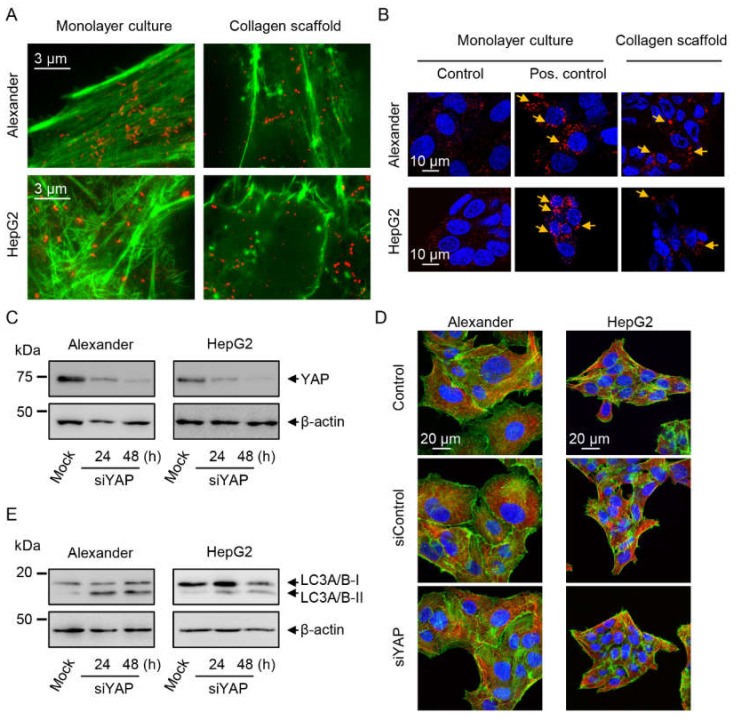
Autophagic flux in cells cultured in collagen scaffolds. (**A**) IXplore SpinSR Olympus super-resolution microscopy of F-actin staining (green) and pmTOR (red). (**B**) Assessment of cellular autophagosome punctae containing LC3-II by confocal microscopy. HepG2 and Alexander cells were grown either in standard monolayer culture or in collagen scaffolds. Cells were fixed and immunostained for nucleus (blue) and LC3 (red). Labeled cells were then imaged using confocal microscopy. Arrows indicate formation of cellular autophagosome punctae. Positive control—serum starvation for 14 h (Alexander cells) and treatment with 10 mM H_2_O_2_ for 30 min (HepG2 cells). (**C**) Downregulation of YAP. Immunoblot analysis for YAP in Alexander and HepG2 cells transfected with YAP siRNA for 24 and 48 h; β-actin—control of equal protein loading. (**D**) Immunostaining analysis for F-actin (green) and tubulin (red) in Alexander and HepG2 cells transfected with YAP siRNA for 48 h. Hoechst 33342 (blue) dye was used to counterstain nuclei. Labeled cells were then imaged by confocal microscopy and the images were processed using ImageJ software (NIH). (**E**) Immunoblot analysis for LC3 in Alexander and HepG2 cells transfected with YAP siRNA for 24 and 48 h; β-actin—control of equal protein loading.

Further, to reveal, in detail, the contribution of pmTOR and cytoskeleton remodeling in mechanically-driven regulation of proliferation, we assessed F-actin remodeling and pmTOR localization under higher resolution using novel super-resolution spinning disk microscopy. It has been shown that upon stimulation with mechanical stimuli, mTOR is associated with actin stress fibers, in turn supporting cell proliferation [88]. Super-resolution imaging confirmed F-actin remodeling in cells grown in collagen scaffolds (Figure 6A). More importantly, this analysis revealed that in cells grown in monolayer culture, pmTOR was localized in vesicle-like inclusions associated with actin stress fibers (Figure 6A). On the contrary, cells grown in collagen scaffolds showed punctuated pmTOR distribution in the cytoplasm (Figure 6A). Taking into account that cells grown in collagen scaffolds displayed low proliferation activity (Figure 2E,F), these data, taken together, indicate that mTOR subcellular translocation modulates cell division.

It is worth noting here that mTOR is a well-known negative regulator of autophagy [77,78]. Thus, we analyzed LC3–phosphatidylethanolamine conjugate (LC3-II) as one of the major autophagic markers by puncta formation in microscopic immunofluorescent analysis [89,90]. Indeed, both Alexander and HepG2 cells cultured in collagen scaffolds led to increased LC3-positive punctae, confirming activated autophagy (Figure 6B). In fact, these data are in line with pmTOR downregulation upon cell culturing in collagen scaffolds (Figure 5A,B).

Summarizing the data until now, we revealed that culturing Alexander and HepG2 cells in collagen scaffolds leads to YAP and pmTOR downregulation and cytosolic translocation, which is accompanied by activated autophagic flux. In turn, this results in a slow proliferation rate of cells and changes in cell size and morphology. In fact, cell phenotypic plasticity has already been shown to be dependent on autophagic flux driven by YAP mechanotransduction [32]. Specifically, it was identified that YAP knockdown leads to an increased autophagosome formation [32]. These data are perfectly in line with our observations.

To further prove the role of YAP in the coordination of cellular shape and mTOR autophagy-related signaling, we downregulated YAP expression. First of all, we checked efficiency of YAP downregulation by siRNA for HepG2 and Alexander cells (Figure 6C). Indeed, YAP downregulation did not impact actin levels (Figure 6C). The highest YAP downregulation by siRNA was detected 48 h post-transfection (Figure 6C). Surprisingly, YAP downregulation did not affect cellular shape and cytoskeleton remodeling (Figure 6D). However, YAP inactivation increased the levels of LC3-II (Figure 6E).

**Figure 7:**
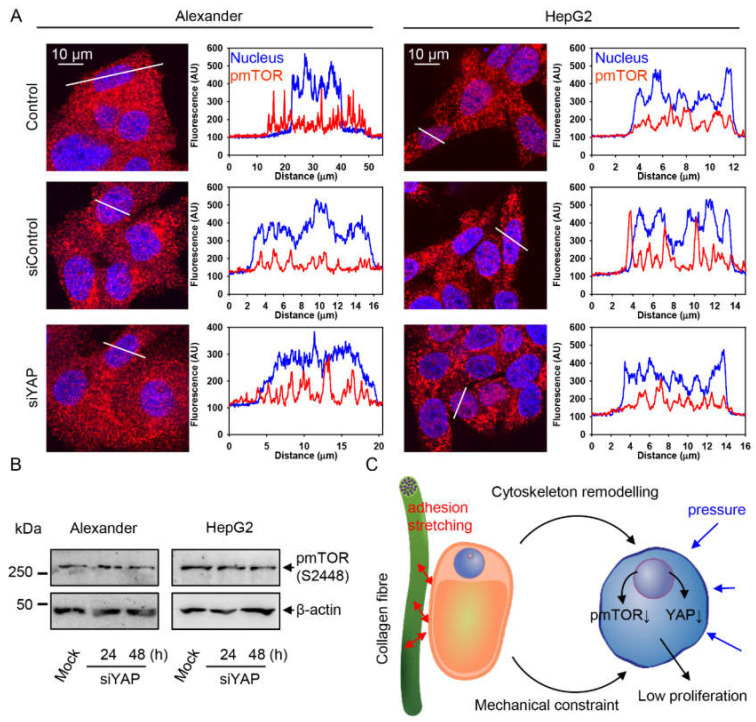
YAP downregulation does not change pmTOR signaling. (**A**) Immunostaining analysis for pmTOR (red) in Alexander and HepG2 cells transfected with YAP siRNA for 48 h. Hoechst 33342 (blue) dye was used to counterstain nuclei. Labeled cells were then imaged by confocal microscopy and the imaged were processed using ImageJ software (NIH). Representative confocal microscopic images and line scans. (**B**) Immunoblot analysis for pmTOR in Alexander and HepG2 cells transfected with YAP siRNA for 24 and 48 h; β-actin—control of equal protein loading. (**C**) Scheme of competing mechanical cues, i.e., adhesion and pressure, that cells experience in collagen scaffolds. Mechanistic explanation of signaling events modulated by mechanical cues; ↓—downregulation.

Interestingly, YAP downregulation did not affect either pmTOR sub-cellular localization (Figure 7A and Supplemental Figure S8) or protein expression level (Figure 7B). By using the downregulation approach, we intended to strengthen the link between YAP downregulation and autophagy promotion. On the other hand, these data imply that siRNA silencing of YAP in a 2D culture is not similar to the downregulation triggered under mechanical cues in a 3D environment. These findings confirm the influence of YAP on controlling autophagy. However, they imply that in collagen scaffolds, cells experience competing mechanical cues (Figure 7C), and the interplay of both YAP and mTOR signaling mediates cellular adaptation and plasticity in a 3D microenvironment.

## 3. Discussion

Recent developments in 3D cell culturing have shown the drawbacks of standard 2D monolayer cultures [33,34,35]. Specifically, standard monolayer cultures bear substantial limitations in predicting cellular responses of liver cancer cell behavior [7,91,92]. In fact, the mechanical properties of standard culturing glass or plastic differ drastically from physiological ECM or collagen scaffold surrogates [93,94,95]. Thus, it was realized that the physical properties of the cellular microenvironment and mechanical forces are critical regulators of fundamental cell properties, such as cell size and shape, plasticity, migration and dormancy [2,3,4,7,96,97]. However, molecular mechanisms regulating those processes have not been fully addressed.

Therefore, here, we present a study that comparatively addresses 3D cellular culture-driven mechanotransduction in liver cancer cells. We propose a model for the organization and distribution of competing mechanical cues within a 3D cell culture microenvironment that controls cell size and shape, plasticity and dormancy of two liver cancer cell lines (Figure 7C).

It is worth noting here that the stiffness of a standard glass or plastic culture is in the GPa range, whereas collagen scaffolds or ECM in vivo are characterized by stiffness ranging from 0.1 to 30 kPa [93,94,95]. Additionally, several studies have shown that cells (including hepatic HepG2 and Huh7 cell lines) dramatically slow down their proliferation when cultured in a soft 2D or 3D microenvironment [7,30,98,99,100]. Therefore, we compared, here, these two extremes, i.e., monolayer cultures on standard stiff glass and 3D cell culturing in soft collagen scaffolds. Our data are in line with previous studies, showing that both HepG2 and Alexander cells slowed down their proliferation dramatically when cultured in collagen scaffolds (Figure 2E,F and Supplemental Figure S4). Despite very slow proliferation, both cell lines were able to substantially populate the collagen scaffolds (Videos S1 and S2), stay viable for 7 days of culture (Figure 2A) and respond normally to ethanol toxicosis (Figure 2D and Supplemental Figure S3).

Furthermore, we observed that both Alexander and HepG2 cells change their morphology while populating collagen pores (Figure 3C,D). In fact, cells close to the center of collagen pores showed a rounded morphology, whereas stretched and flattened cells were predominant on collagen fibers (Figure 3C,D). It is known that the YAP pathway is involved in maintaining cell density and shape [20,21]. Indeed, nuclear localization of YAP was predominant in cells located at the edge of collagen pores, whereas cells distributed closer to the center of collagen pores showed cytosolic YAP localization (Figure 4C–E). Thus, we tentatively proposed that the collagen scaffold microenvironment offers, to cells, distinct competing mechanical cues in the center and at the edge of collagen pores. Cells close to collagen fibers are exposed to adhesion and stretching, contrary to the center of collagen pores where cells experience pressure from neighboring cells (Figure 7C). We indirectly confirmed this hypothesis by assessing F-actin distribution within cells. Protrusions resembling filopodia were identified in cells at the edge of collagen pores (Figure 4A). Additionally, bright spots of F-actin stress fibers were observed in cells located closer to the center of collagen pores (Figure 4B). In fact, a recent study showed that external pressure above 20 Pa exerted on cells promoted F-actin reorganization and led to YAP cytoplasmic translocation [101]. This study supports our findings on YAP cytoplasmic translocation (Figure 4C,D).

Further, it was shown that mechanical stress modulates mTOR signaling [30,75,76]. Overall, the total levels of the phosphorylated form of mTOR were significantly lower in cells cultured in soft collagen scaffolds in comparison to the monolayer culture on standard stiff glass (Figure 5A,B). mTOR signaling downregulation was accompanied by activated autophagy in the cells grown in collagen scaffolds (Figure 6B). Our findings are in line with a study showing that mechanical inputs regulate autophagy [32]. Moreover, we found that mTOR sub-cellular distribution followed YAP cytoplasmic translocation (Figure 4C,D and Figure 5C,D). Interestingly, it was shown that mTOR activity follows matrix stiffness in vivo in both mouse and human breast tumors; i.e., a stiff ECM activates mTOR and supports proliferation, whereas a soft ECM leads to mTOR inhibition and lower proliferating activity [30]. Our data are in line with those findings and tentatively indicate that in vivo tumor heterogeneity could be potentially mimicked by collagen scaffold cultures. Furthermore, on the one hand, mTOR regulates YAP activity [28], and on the other hand, YAP modulates mTOR signaling [29]. Our study reveals that YAP and mTOR signaling are interconnected in complex 3D microenvironments. The interplay of those signals regulates the proliferation of liver cancer cells. The findings presented here describe a convergence of YAP and mTOR signaling pathways, which are modulated by mechanical cues.

## 4. Materials and Methods

### 4.1. Chemicals and Antibodies

All chemicals and antibodies used for this study are compiled in Tables S1–S3 of the Supplemental Materials, including manufacturers, catalogue numbers and dilutions.

### 4.2. Synthesis and Characterization of Collagen Scaffolds

The porous collagen scaffolds were prepared by the cryostructuring technique [46]. The lyophilized collagen (VUP Medical, a.s., Brno, Czech Republic, dry basis min. 80%) was solubilized in acetic acid (final concentration 0.1%) at 4 °C, then centrifuged at 2000 g for 30 min at 4 °C to achieve the final collagen concentration around 8 mg/mL in a paste-like sediment. The former one was poured into plastic molds (diameter 3 mm) that were placed in a freezer and incubated at −20 °C for 24 h. Then, the frozen samples were immersed into 96% ethanol pre-cooled to −20 °C and kept with periodical shaking for 2 days with the ethanol changed every day. Finally, the scaffolds were transferred into a 1% N-(3-Dimethylaminopropyl)-N′-ethylcarbodiimide (EDC) hydrochloride (Sigma) ethanol solution, pre-cooled to −20 °C, for another 24 h. After thawing, scaffolds were rinsed with ethanol and dH_2_O and stored in the 70% ethanol until use. Before use, the scaffolds were extensively washed with phosphate-buffered saline (PBS) and culture medium.

Characterization of the scaffolds’ rheological/viscoelastic properties was performed by the Anton Paar modular compact rheometer MCR 92, i.e., plate–plate crosshatched geometry (Anton Paar Ltd., St Albans, UK). For determination of the storage modulus G’ and loss modulus G’’, the oscillatory strain sweep mode with frequency 1 Hz was used.

In order to analyze the morphology of the prepared collagen scaffolds in a native liquid environment, scaffolds were labelled with fluorescent Col-F collagen-binding reagent (ImmunoChemistry Technologies, LLC, Bloomington, USA). Labelled scaffolds were imaged using the spinning disk confocal microscope IXplore SpinSR (Olympus, Tokyo, Japan). ImageJ software (NIH, Bethesda, MD, USA) was used for image processing and quantification of the pore size and fiber width of collagen scaffolds.

To analyze the diffusivity of the collagen scaffolds, we utilized the fluorescence correlation spectroscopy (FCS) technique. Measurements were performed using an inverted confocal fluorescence microscope, Olympus IX71 (Olympus, Hamburg, Germany), equipped with a single photon counting unit MicroTime 200 (PicoQuant, Germany). Excitation lasers of 470 and 640 nm (PicoQuant, Germany), operating at 20 MHz, were used to illuminate a sample through the water-immersion objective (1.2 NA, 60x) (Olympus). Fluorescence signal was gathered through the main dichroic mirror (Z473/635, Chroma, Rockingham, VT) and a 50-μm pinhole. The second dichroic mirror (620 nm, Chroma, Rockingham, VT) was used to separate the red fluorescence of Alexa 647 from the blue fluorescence of lipid analog and dextrans. Two single-photon avalanche diodes were used to collect red and blue fluorescence simultaneously. All FCS measurements were performed at 25 °C in 8-well μ-Slides (Ibidi, Germany). Since the particles were found to adsorb to the glass, μ-Slides with plastic bottom were used, to which adsorption was not observed. Calibration measurements were performed using Atto 488 (Atto-tec, Germany).

The measured data were fitted using standard 3D diffusion model in Symphotime 64 software (PicoQuant, Germany). A fast diffusion component was fixed based on the measurement in bulk solution at 0.035 ms for Alexa647, 0.11 ms for dextran 3000 and 0.185 ms for dextran 10,000. Fluorescence decay data were used to correct the noise.

### 4.3. Cell Lines and 3D Culturing

The human hepatoblastoma HepG2 cell line (American Type Culture Collection, ATCC) and human hepatocellular carcinoma cell lines Alexander (PLC/PRF/5, ATCC) were used in this study. Cells were cultured in EMEM medium (ATCC) supplemented with 10% fetal bovine serum (FBS, Thermo Fisher Scientific, Waltham, MA, USA) and 1% penicillin/streptomycin (Thermo Fisher Scientific, Waltham, MA, USA). Cell cultures were cultivated in a humidified 5% CO_2_ atmosphere at 37 °C. Cell culture medium was replaced once a week. Cells seeded on 35-mm wide standard glass-bottom dishes (Cellvis, Sunnyvale, CA, USA) served as monolayer culture model.

Alexander and HepG2 cells were seeded into collagen scaffolds placed in 12-well plates. Approximately 10^5^ of Alexander or HepG2 cells in 30-µL volume were seeded into collagen scaffolds. After 1 h, a fresh medium was added in order to cover up the surface of the scaffolds. For each analysis, cells had grown in collagen scaffolds for 7 days in a humidified 5% CO_2_ atmosphere at 37 °C. Medium was changed each second day.

### 4.4. Assessment of Cell Viability

We utilized the fluorescent LIVE/DEAD^®^ Viability/Cytotoxicity Kit (Thermo Fisher Scientific, Waltham, MA, USA). The assay is based on detection of the uniform green fluorescence of calcein-AM retained within live cells and identification of ethidium homodimer, which labels, with bright red fluorescence, the nuclei of dead cells [48,102,103]. Labeled cells were imaged using the spinning disk confocal microscope IXplore SpinSR (Olympus, Tokyo, Japan).

To verify the responsiveness of cells grown in collagen scaffolds, we subjected cells to ethanol toxicosis. HepG2 and Alexander cells were grown either in s standard monolayer culture or in collagen scaffolds. Afterwards, cells were labeled with CellMask™ Green (Thermo Fisher Scientific, Waltham, MA, USA), as a membrane stain, and propidium iodide (Thermo Fisher Scientific, Waltham, MA, USA), as a dead cell stain. Hoechst 33342 (Thermo Fisher Scientific, Waltham, MA, USA) dye was used to counterstain nuclei. Control cells were untreated. As a positive control, cells were treated with 20% ethanol for 60 min. Labeled cells were then imaged by confocal microscopy. ImageJ software (NIH, Bethesda, MD, USA) was used for image processing and 3D reconstruction.

### 4.5. Cell Proliferation Analysis

To analyze cell proliferation, we utilized three different approaches. Firstly, cells were grown in either collagen scaffolds or monolayer culture for 1, 2, 3 and 4 days under standard conditions (37 °C, 5% CO_2_). Nuclei were stained with Hoechst 33342 (Thermo Fisher Scientific, Waltham, US). The stained nuclei of cells were imaged using the spinning disk confocal microscope IXplore SpinSR (Olympus, Tokyo, Japan). Relative cell number was calculated as Total cell number/Total cell number at day 0, utilizing ImageJ software (NIH, Bethesda, MD, USA). Cell counting was carried out for 5 fields of view per sample. Three samples per surface were set aside for cell proliferation measurements, and the expressed values are the mean ± standard error of the mean (SEM). Additionally, we detected levels of PCNA using immunoblotting and of Ki-67 using immunofluorescence approaches to validate proliferation of cells.

### 4.6. Cell Extracts and Immunoblot Analysis

To assess different proteins’ expression, we used an immunoblot analysis. We obtained cell extracts using the lysis radioimmunoprecipitation assay (RIPA) buffer (Millipore, Burlington, VT, USA) in accordance with the manufacturer’s instructions and our verified protocol [86,104,105]. Then, aliquots of whole-cell lysates containing equal amounts of protein were prepared. Proteins were separated utilizing SDS-PAGE electrophoresis, then transferred to polyvinylidene fluoride (PVDF) membranes. Membrane blocking was performed using either 5% (*w/v*) fat-free dried milk or, alternatively, with 5% (*w/v*) bovine serum albumin (BSA) for 1h. Afterwards, membranes were incubated with various specific primary antibodies summarized in Section 4.1 at 4 °C overnight. Chemiluminescence signals were detected using the imaging system GBOX CHEMI XRQ (Syngene, Synoptics group, Cambridge, UK) as described [104,106]. The acquisition software GeneTools (Syngene, Synoptics group, Cambridge, UK) was used for chemiluminescence detection. Densitometric quantification of blots was performed using GeneTools quantification software (Syngene, Synoptics group, Cambridge, UK).

### 4.7. Immunofluorescence

Cells were grown in either collagen scaffolds or monolayer culture under standard conditions (37 °C, 5% CO_2_). Afterwards, cells were either life-labelled or fixed and immunostained utilizing antibodies and/or fluorescent probes summarized in Section 4.1. Cells were fixed with 4% paraformaldehyde in PBS pH 7.4 at room temperature for 10 min. Samples were permeabilized using 0.5% Triton X-100 before staining. Immunofluorescence staining was performed on fixed cells using primary antibodies against different proteins, summarized in Section 4.1, and Alexa Fluor 568-conjugated secondary antibody (Thermo Fisher Scientific, Waltham, MA, USA). Dilutions and catalogue numbers of the primary antibodies used are given in summarized in Section 4.1. Stained cells were imaged using the spinning disk confocal microscope IXplore SpinSR (Olympus, Tokyo, Japan). ImageJ software (NIH, Bethesda, MD, USA) was used for image processing and quantification.

### 4.8. Confocal Microscopy

For high-quality confocal images, we utilized the brand new high-resolution spinning disk confocal system IXplore SpinSR (Olympus, Tokyo, Japan) [107,108]. The system was equipped with an inverted microscope (IX83; Olympus, Tokyo, Japan) and a spinning disc confocal unit (CSUW1-T2S SD; Yokogawa, Musashino, Japan). Fluorescence images were obtained through either a 100× silicone immersion objective (UPLSAPO100XS NA 1.35 WD 0.2 silicone lens, Olympus, Tokyo, Japan) or a 20× objective (LUCPLFLN20XPH NA 0.45 air lens, Olympus, Tokyo, Japan). Fluorophores were excited by appropriate wavelengths, namely a 405-nm laser diode (50 mW), a 488-nm laser diode (100 mW) and a 561-nm laser diode (100 mW). Confocal images were acquired at a definition of 2048 × 2048 pixels. The images were passed through appropriate emission filters (BA420-460; BA575IF; BA510-550; Olympus, Tokyo, Japan) and captured simultaneously by two digital CMOS cameras ORCA-Flash4.0 V3 (Hamamatsu, Hamamatsu City, Japan). Fluorescence confocal images were taken with the acquisition software cellSens (Olympus, Tokyo, Japan). ImageJ software (NIH, Bethesda, MD, USA) was used for image processing and quantification of the following parameters: cell and nuclei sizes; collagen pore size; collagen fiber width; cell number; fluorescence line scans. Quantitative image analysis was performed by randomly selecting ∼ 5–10 visual fields per sample, using the same setting parameters (i.e., spinning disk speed, laser power and offset gain).

### 4.9. Spinning Disk Super-Resolution Microscopy

Cells were grown in either collagen scaffolds or monolayer culture under standard conditions (37 °C, 5% CO_2_). Afterwards, cells were fixed, permeabilized and immunostained utilizing antibody against pmTOR; dilution and specification of the antibody is summarized in Section 4.1. F-actin was labeled using the ActinGreen™ 488 ReadyProbes™ reagent (Thermo Fisher Scientific, Waltham, MA, USA). In order to perform super-resolution imaging of actin cytoskeleton and pmTOR, we utilized the novel IXplore SpinSR Olympus super-resolution imaging system (Olympus, Tokyo, Japan). The system was equipped with an inverted microscope (IX83; Olympus, Tokyo, Japan), a spinning disc confocal unit (CSUW1-T2S SD; Yokogawa, Musashino, Japan) and a super-resolution unit (CS-S-OSR-V1 Super Resolution, Olympus, Tokyo, Japan). Fluorescence super-resolution images were obtained through a 100× silicone immersion objective (UPLSAPO100XS NA 1.35 WD 0.2 silicone lens, Olympus, Tokyo, Japan). ActinGreen™ 488 and pmTOR were excited by a 488-nm laser diode (100 mW) and a 561-nm laser diode (100 mW), respectively. The images were acquired at a definition of 1024 × 1024 pixels by passing through appropriate emission filters (BA575IF; BA510-550; Olympus, Tokyo, Japan) and were captured by two digital CMOS cameras ORCA-Flash4.0 V3 (Hamamatsu, Hamamatsu City, Japan). The super-resolution images were acquired using the software cellSens (Olympus, Tokyo, Japan). ImageJ software (NIH, Bethesda, MD, USA) was used for image processing.

### 4.10. Transient Transfection with siRNA

Cells were seeded onto 12-well plates at a density of 400,000 cells per well. The day after, cells were transfected with 1.5 µg of YAP1 Silencer Select per well using Lipofectamine 3000 transfection reagent (Thermo Fisher Scientific, Waltham, MA, USA). As recommended by the manufacturer, the siRNA was premixed in OptiMEM medium (Thermo Fisher Scientific, Waltham, MA, USA), incubated with diluted Lipofectamine Reagent and then the complex of lipid–siRNA was added to the cells. The transfected cells were harvested at 24 and 48 h for protein analysis. For the immunofluorescence analysis, the cells were seeded onto 6-channel Ibidi μ-Slides (Ibidi, Martinsried, Germany) at a density of 15,000 cells per channel and transfected the next day with 150 ng of YAP1 Silencer Select or negative control Silencer. Then, 48 h after transfection, cells were stained as described in Section 4.7.

### 4.11. Quantification of YAP Subcellular Localization

To measure YAP localization in the nucleus, we adopted previously published methodology [109]. Cells were grown in either collagen scaffolds or monolayer culture under standard conditions (37 °C, 5% CO_2_). Afterwards, cells were fixed, permeabilized and immunostained utilizing antibody against YAP; dilution and specification of the antibody is summarized in Section 4.1. Nuclei were stained with Hoechst 33342 (Thermo Fisher Scientific, Waltham, MA, USA). Stained cells were imaged using the spinning disk confocal microscope IXplore SpinSR (Olympus, Tokyo, Japan). ImageJ software (NIH, Bethesda, MD, USA) was used for image processing and quantification. Eleven z-stack images covering 5 μm were acquired. For each cell, we calculated the nuclear/cytosolic YAP ratio as: Nuc/Cyt YAP ratio = Nuc_IntDen_ YAP/(Cell_IntDen_ YAP—Nuc_IntDen_ YAP), where Nuc_IntDen_ YAP is the integrated YAP intensity in the nucleus and Cell_IntDen_ YAP is the integrated YAP intensity of the whole cell.

### 4.12. Statistical Analysis

Quantitative results are presented as mean ± SEM. The sample size determination was assessed utilizing a statistical method described in [110], taking into assumption 95% confidence level and 0.9 statistical power. The statistical significance of differences between the groups was determined using ANOVA with subsequent application of either the Newman–Keuls test or the Mann–Whitney U test. All statistical analyses were performed using MaxStat Pro 3.6. Differences were considered statistically significant at (*) *p* <  0.05.

For a quantitative analysis of the images, we utilized the published guidance for quantitative confocal microscopy [111,112]. Images from three independent experiments were subjected to quantitative analysis. In each experiment, 10 randomly selected fields from each sample were imaged. In order to determine sample size, we utilized a previously described statistical method [110]. According to this method, the sample size for 95% confidence level and 0.9 statistical power corresponds to 30. Thus, at least 30 randomly selected cells were used in fluorescence microscopy quantification.

## 5. Conclusions

In conclusion, the present study not only demonstrated control of liver tumor cell plasticity by physical cues originated in a 3D microenvironment but also identified the participation of the YAP–mTOR axis in mechanically-driven regulation of cell proliferation. Here, we showed the interplay of YAP and mTOR signals under mechanical constraint in liver tumor cells. We found that collagen scaffolds exert distinct competing mechanical cues on cultured cells. Adhesion and pressure modulate YAP and mTOR activity and subcellular localization. Our results support a recently published paradigm that suggests involvement of YAP signaling in regulation of autophagy [32]. The findings presented here provide an insight into YAP–mTOR-driven mechanotransduction regulation under physical cues.

## Figures and Tables

**Table 1:** Diffusion times measured in collagen scaffolds using fluorescence correlation spectroscopy. The results obtained in the pores and in the collagen matrix are compared.

Sample Description	Diffusion Times (ms) and Fractions ^1^	
	Scaffold Pores	Collagen Matrix
Alexa 647 in collagen scaffold (sample 1)	0.35 ± 0.06 (26 ± 9)%	0.63 ± 0.19 (45 ± 6)%
Alexa 647 in collagen scaffold (sample 2)	0.32 ± 0.06 (17 ± 3)%	0.60 ± 0.08 (38 ± 4)%
Alexa 647 in collagen scaffold (sample 3)	0.22 ± 0.02 (21 ± 2)%	0.43 ± 0.15 (45 ± 5)%
Alexa 647 in collagen scaffold (sample 4)	0.86 ± 0.37 (14 ± 2)%	0.91 ± 0.33 (42 ± 2)%
Dextran 3000-Alexa 488 (sample 3)	1.32 ± 0.78 (27 ± 17)%	3.39 ± 1.27 (47 ± 11)%
Dextran 3000-Alexa 488 (sample 3)	1.49 ± 0.70 (23 ± 8)%	44.65 ± 17.80 (8 ± 1)%
Dextran 10,000-Alexa 488 (sample 4)	0.57 ± 0.51 (28 ± 3)%	2.02 ± 0.40 (25 ± 4)%
Dextran 10,000-Alexa 488 (sample 4)	0.81 ± 0.44 (16 ± 4)%	3.35 ± 0.45 (37 ± 6)%
DOPC-Bodipy in POPC liposomes (sample 1)	9.46 ± 4.96	90.74 ± 56.49
DOPC-Bodipy in POPC liposomes (sample 2)	1.86 ± 0.12	125.46 ± 99.03

^1^ The presented results are averages from at least four points from diffusion maps (see Supplemental Figure S1) and are presented separately for different locations in collagen scaffold samples. Uncertainty is given as standard error. The results show both longer diffusion time and its fraction, in the case of two-component fitting, or mean diffusion time, in the case of single component model. 1-Palmitoyl-2-oleoyl-sn-glycero-3-phosphocholine (POPC), 1,2-dioleoyl-sn-glycero-3-phosphocholine (DOPC).

## Supplementary Materials

The following are available online at https://www.mdpi.com/1424-8247/13/12/430/s1, Figure S1: Analysis of the diffusivity of Alexa 647, Alexa 488-dextrans and liposomes labeled with Bodipy-lipid analog in collagen scaffolds assessed by Fluorescence Correlation Spectroscopy (FCS); Figure S2: Hepatic cell size and plasticity under culturing in collagen scaffolds confocal analysis; Figure S3: Analysis of ethanol toxicosis of cells grown in collagen scaffolds; Figure S4: Immunofluorescence confocal microscopy of Ki-67-stained HepG2 and Alexander cells; Figure S5: 3D reconstruction of cytoskeleton remodeling under culturing in collagen scaffolds; Figure S6: Maximum intensity projections of cytoskeleton remodeling under culturing in collagen scaffolds; Figure S7: Sub-cellular localization of YAP under culturing in collagen scaffolds; Figure S8: Sub-cellular distribution of pmTOR upon YAP siRNA transfection. Table S1: List of chemical probes used in the study; Table S2: List of fluorescent probes used in the study; Table S3: List of antibodies used in the study; Uncropped immunoblot scans. Video S1: 3D reconstruction of Alexander cells grown in collagen scaffolds; cell membranes were labeled with CellMask™ Green (green). Hoechst 33342 (blue) dye was used to counterstain nuclei. Labeled cells were then imaged by confocal microscopy; Video S2: 3D reconstruction of HepG2 cells grown in collagen scaffolds; cell membranes were labeled with CellMask™ Green (green). Hoechst 33342 (blue) dye was used to counterstain nuclei. Labeled cells were then imaged by confocal microscopy.
