# Supplementary material for: Hepatic Tumor Cell Morphology Plasticity under Physical Constraints in 3D Cultures Driven by YAP–mTOR Axis
Source: Pharmaceuticals (Basel). 2020 Nov 28;13(12):430. doi: 10.3390/ph13120430 (PMC7759829; doi:10.3390/ph13120430)
Supplement: Supplementary file 1 [file pharmaceuticals-13-00430-s001.zip › Supplementary data.pdf]

Supplementary material to

Hepatic Tumor Cell Morphology Plasticity Under Physical Constraints in 3D Cultures driven by YAP-mTOR Axis

Adam Frtús [1], Barbora Smolková [1], Mariia Uzhytchak [1], Mariia Lunova [1,2], Milan Jirsa [2], Martin Hof [3], Piotr Jurkiewicz [3], Vladimir I. Lozinsky [4], Lucie Wolfová [5,6], Yuriy Petrenko [5], Šárka Kubinová [1,5], Alexandr Dejneka [1,*] and Oleg Lunov [1,*]

[1] Department of Optical and Biophysical Systems, Institute of Physics of the Czech Academy of Sciences, Prague, 18221, Czech Republic; frtus@fzu.cz (A.F.); smolkova@fzu.cz (B.S.); uzhytchak@fzu.cz (M.U.); dejneka@fzu.cz (A.D.); lunov@fzu.cz (O.L.)

[2] Institute for Clinical & Experimental Medicine (IKEM), Prague, 14021, Czech Republic; mariialunova@googlemail.com (M.L.); miji@ikem.cz (M.J.)

[3] J. Heyrovský Institute of Physical Chemistry of the Czech Academy of Sciences, Prague, 18223, Czech Republic; martin.hof@jh-inst.cas.cz (M.H.); piotr.jurkiewicz@jh-inst.cas.cz (P.J.)

[4] A.N. Nesmeyanov Institute of Organoelement Compounds, Russian Academy of Sciences, Vavilov Street, 28, 119991 Moscow, Russia; loz@ineos.ac.ru (V.I.L.)

[5] Department of Biomaterials and Biophysical Methods, Institute of Experimental Medicine of the Czech Academy of Sciences, Prague, 14220, Czech Republic; yuriy.petrenko@iem.cas.cz (Y.P.); sarka.kubinova@iem.cas.cz (S.K.)

[6] Department of Tissue Engineering, Contipro a.s., Dolni Dobrouc, Czech Republic; lucie.wolfova@iem.cas.cz (L.W.)

[*] Correspondence: dejneka@fzu.cz; Tel.: +420-2660-52141; lunov@fzu.cz; Tel.: +420-2660-52131

Supplementary Tables

Table S1 List of chemicals probes used in the study.

Reagent	Manufacturer	Catalogue
PBS	Gibco	10010015
Triton-X100	PanReac AppliChem	A4975,0100
Micro BCA Proein Assay Kit	Thermo Fisher Scientific	23235
RIPA buffer	Millipore	20188
Protease Inhibitor Cocktail	Sigma Aldrich	P8340-1ML
Phosphatase Inhibitor Cocktail 3	Sigma Aldrich	P0044-1ML
Paraformaldehyde	VWR	100503-917
YAP1 Silencer Select	Ambion	4392421
Negative control #1 siRNA Silencer Select	Ambion	4390844
Lipofectamine 3000 Reagent	Thermo Fisher Scientific	L3000-008
Opti-MEM	Thermo Fisher Scientific	31985-062
BSA	Sigma Aldrich	2153
Blotting-Grade Blocker	Bio-Rad	170-6404
Clarity Max ECL Western Blotting Substrate	Bio-Rad	1705062

Table S2 List of fluorescent probes used in the study.

Reagent	Manufacturer	Catalogue
CellMask orange	Thermo Fisher Scientific	C10045
CellMask green	Thermo Fisher Scientific	C37608
Propidium Iodide	Thermo Fisher Scientific	R37108
Hoechst 33342	Thermo Fisher Scientific	62249
ColF	ImmunoChemistry Technologies	6346
ActinGreen™ 488 ReadyProbes™ Reagent	Thermo Fisher Scientific	R37110

Table S3 List of antibodies used in the study.

Antibody	Clone/catalogue number	Dilution		Manufacturer
		WB	IF	
Anti-YAP	D8H1X/14074	1: 1000	1:100	Cell Signaling Technology
Anti-LC3A/B	D3U4C/12741	1: 1000	1:100	Cell Signaling Technology
Anti-pmTOR	Ser2448/2971S	1: 1000	1:100	Cell Signaling Technology
Anti- β-Tubulin	D2N5G/ 15115	1: 1000	1:100	Cell Signaling Technology
Anti-β-actin	8H10D10/ 3700	1: 1000	N.A.	Cell Signaling Technology
Anti-Ki67	Ab15580	N.A.	1: 1000	Abcam
Anti-PCNA	EPR3821/ab92552	1: 1000	N.A.	Abcam
Anti-GAPDH	ab226408	1:1000	N.A.	Abcam
Anti-GAPDH	ABS16	1:1000	N.A.	Merck

Anti- mouse-HRP	1858413	1:10 000	N.A.	Pierce Biotechnology
Anti-rabbit- HRP	1858415	1:10 000	N.A.	Pierce Biotechnology
AlexaFluor 568 goat anti-rabbit IgG	A-11011	N.A.	1:1000	Thermo Fisher Scientific

NA – not applicable; WB – western blot; IF – immunofluorescence.

Supplementary Figures

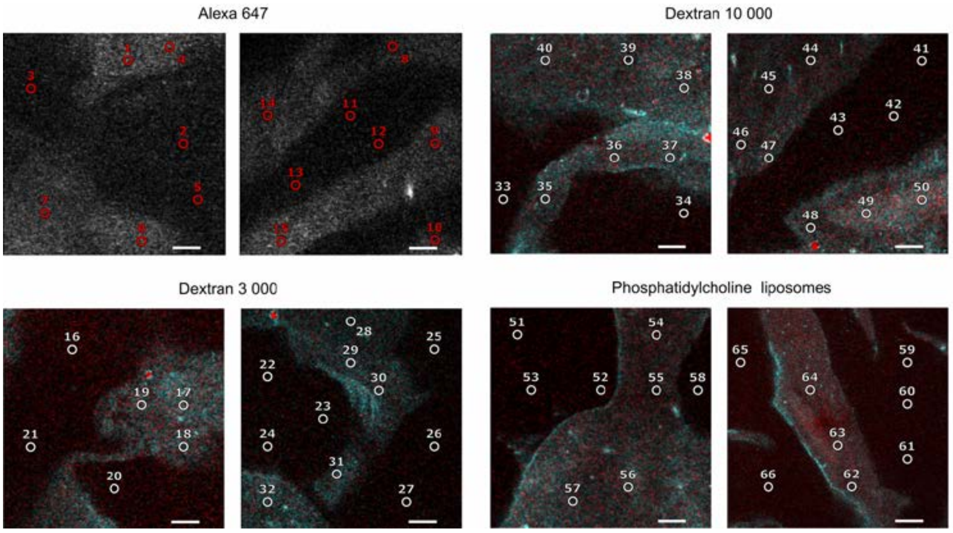

Mean diffusion times	33. 1.07 ms (25%)
	34. 4.63 ms (31%)
**1. 0.44 ms (51%)**	**35. 0.74 ms (43%)**
2. 0.34 ms (16%)	**36. 1.43 ms (20%)**
3. 0.45 ms (12%)	**37. 1.68 ms (24%)**
**4. 0.45 ms (57%)**	**38. 2.22 ms (21%)**
5. 0.22 ms (24%)	**39. 3.50 ms (16%)**
**6. 0.86 ms (51%)**	**40. 2.54 ms (28%)**
**7. 1.23 ms (41%)**	41. 0.89 ms (21%)
**8. 0.85 ms (30%)**	42. 1.52 ms (8%)
**9. 0.72 ms (27%)**	43. 7.19 ms (3%)
**10. 0.49 ms (41%)**	**44. 4.71 ms (42%)**
11. 0.37 ms (13%)	**45. 1.66 ms (65%)**
12. 0.22 ms (23%)	**46. 2.40 ms (35%)**
13. 0.38 ms (15%)	**47. 2.85 ms (28%)**
**14. 0.52 ms (53%)**	**48. 3.77 ms (18%)**
**15. 0.43 ms (40%)**	**49. 3.20 ms (42%)**
	**50. 4.83 ms (31%)**
16. 2.69 ms (11%)	
**17. 7.12 ms (42%)**	51. 1.77 ms (100%]
**18. 2.54 ms (39%)**	52. 1.64 ms (100%]
**19. 2.42 ms (28%)**	53. 1.61 ms (100%]
20. 1.25 ms (10%)	**54. 224 ms (100%**
21. 1.49 ms (79%)	**55. 26.4 ms (100%**
22. 7.99 ms (2%)	**56. ? (> 5 s)**
23. 3.49 ms (4%)	**57. ? (> 5 s)**
24. 0.98 ms (16%)	58. 2.20 ms (100%]
25. 16.10 ms (2%)	59. 2.70 ms (100%]
26. 1.19 ms (38%)	60. 23.6 ms (100%]
27. 0.30 ms (33%)	61. 10.2 ms (100%]
**28. 173.97 ms (5%)**	**62. 47.6 ms (100%**
**29. 88.04 ms (6%)**	**63. 47.0 ms (100%**
**30. 58.12 ms (7%)**	**64. 258 ms (100%**
**31. 23.89 ms (10%)**	65. 2.44 ms (100%]
**32. 8.54 ms (9%)**	66. 9.12 ms (100%]

Figure S1. Analysis of the diffusivity of Alexa 647, Alexa 488-dextrans and liposomes labeled with Bodipy-lipid analog in collagen scaffolds assessed by Fluorescence Correlation Spectroscopy (FCS). Pseudocolor images show Alexa 647 fluorescence in red and the fluorescence of Alexa 488-dextrans and Bodipy-lipid in cyan. Diffusion parameter obtained for the selected points are given in the table on the right hand side.

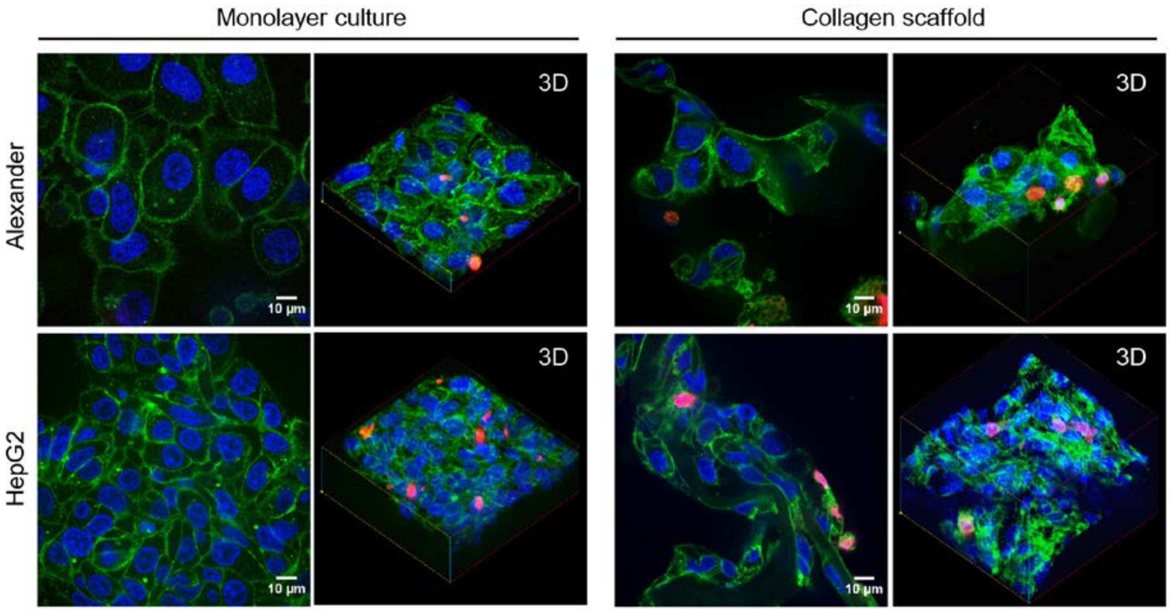

Figure S2. Hepatic cell size and plasticity under culturing in collagen scaffolds confocal analysis. HepG2 and Alexander cells were grown either in standard monolayer culture or in collagen scaffolds. Cell membranes were labeled with CellMask™ Green (green) and propidium iodide (red), as dead cell stain. Hoechst 33342 (blue) dye was used to counterstain nuclei. Labeled cells were then imaged by confocal microscopy, and the images were processed using ImageJ software (NIH). 3D reconstruction was done in ImageJ software (NIH).

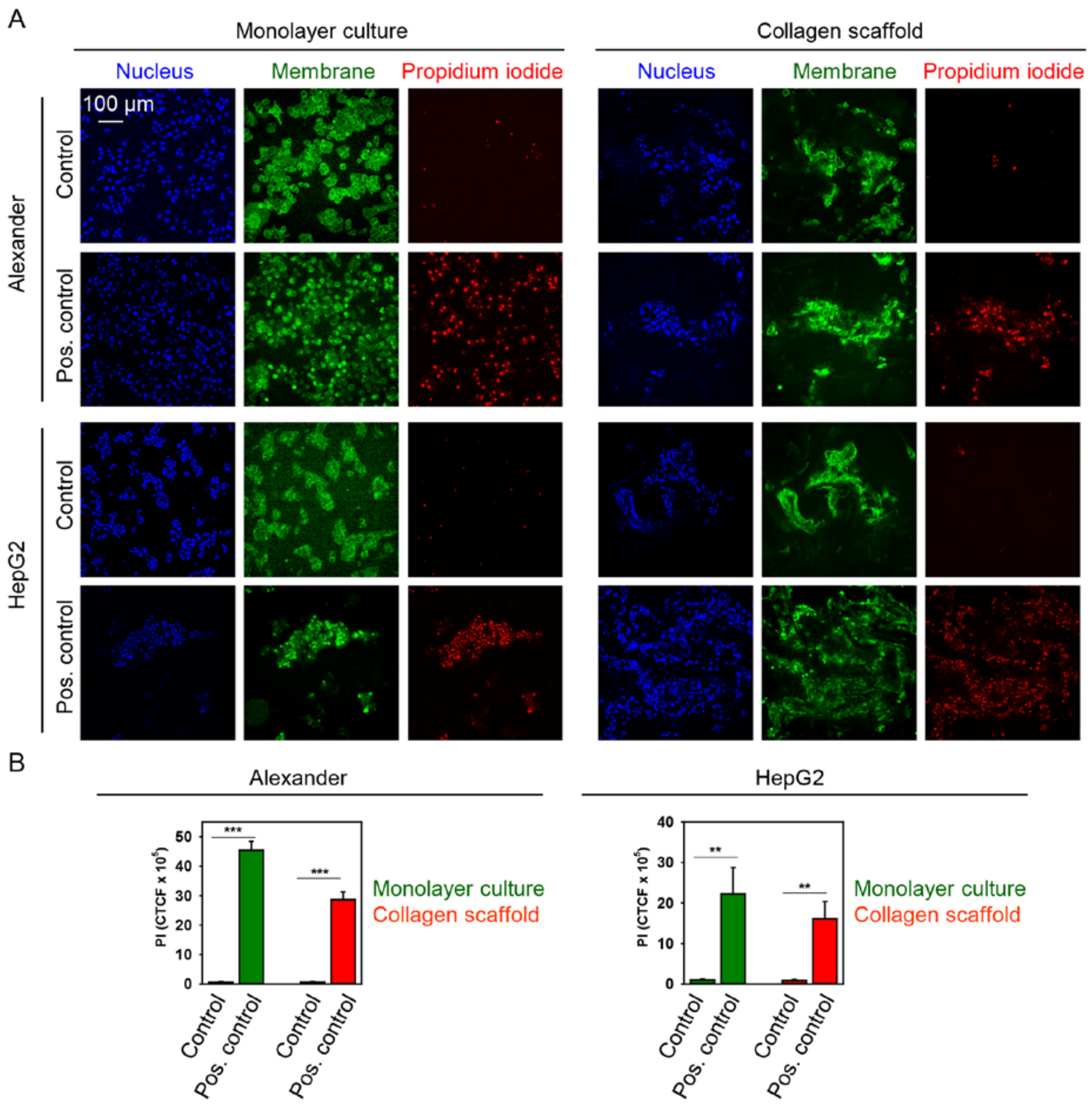

Figure S3. Analysis of ethanol toxicosis of cells grown in collagen scaffolds. HepG2 and Alexander cells were grown either in standard monolayer culture or in collagen scaffolds. Cell were labeled with CellMask™ Green (green), as membrane stain, and propidium iodide (PI, red), as dead cell stain. Hoechst 33342 (blue) dye was used to counterstain nuclei. Control cells were untreated. As a positive control, cells were treated with 20% ethanol for 60 min. Labeled cells were then imaged by confocal microscopy. ImageJ software (NIH) was used for image processing (**A**) and quantification of PI (**B**).

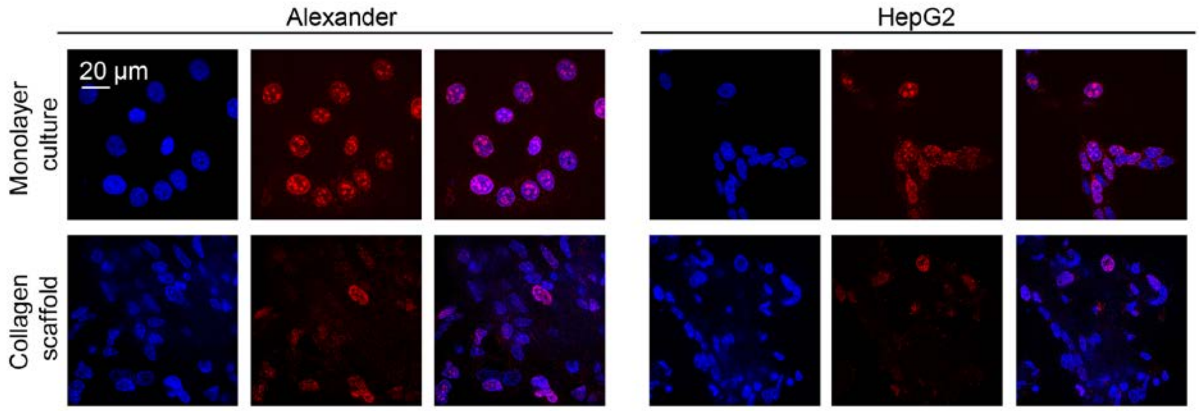

Figure S4. Immunofluorescence confocal microscopy of Ki-67 stained HepG2 and Alexander cells. Cells were grown either in standard monolayer culture or in collagen scaffolds, then fixed, permeabilized and stained for Ki-67 (red). Hoechst 33342 (blue) dye was used to counterstain nuclei. Labeled cells were then imaged by confocal microscopy, and the images were processed using ImageJ software (NIH).

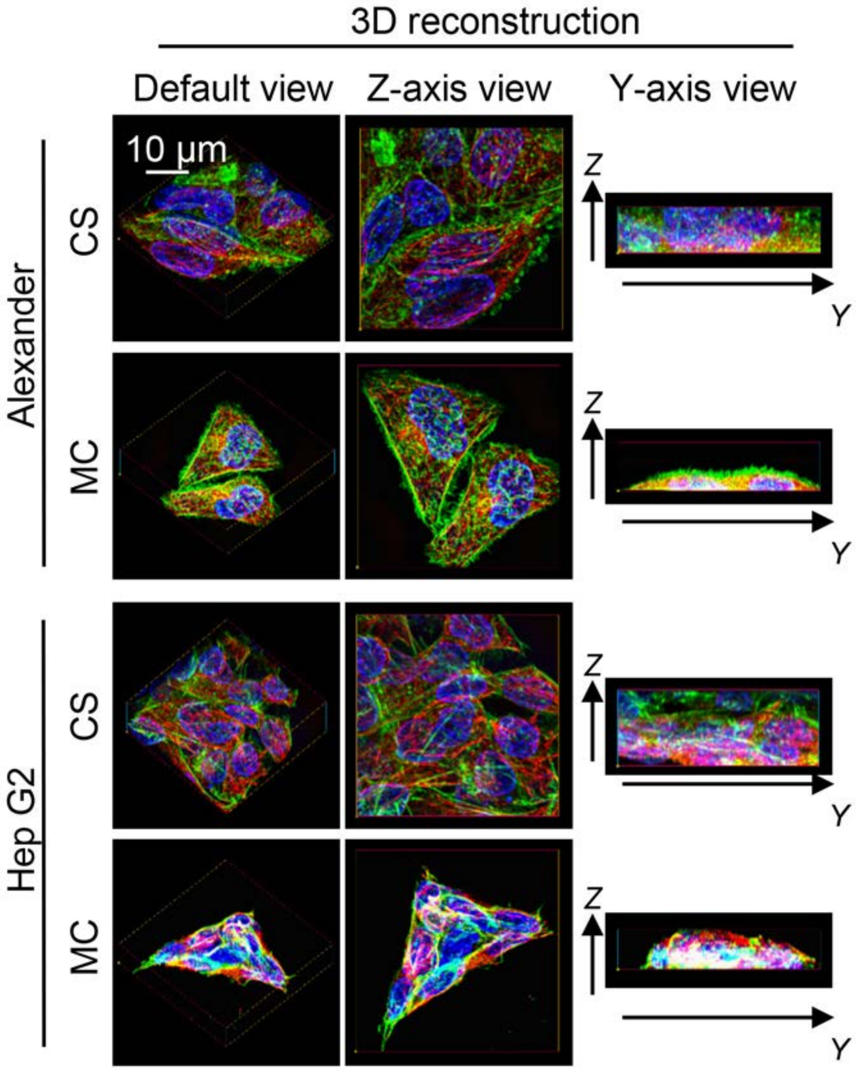

Figure S5. 3D reconstruction of cytoskeleton remodeling under culturing in collagen scaffolds. Cells were grown either in standard monolayer culture or in collagen scaffolds,

then fixed, permeabilized and stained for F-actin (green) and tubulin (red). Hoechst 33342 (blue) dye was used to counterstain nuclei. Labeled cells were then imaged by confocal microscopy, and the image was processed using ImageJ software (NIH). 3D reconstruction was done in ImageJ software (NIH).

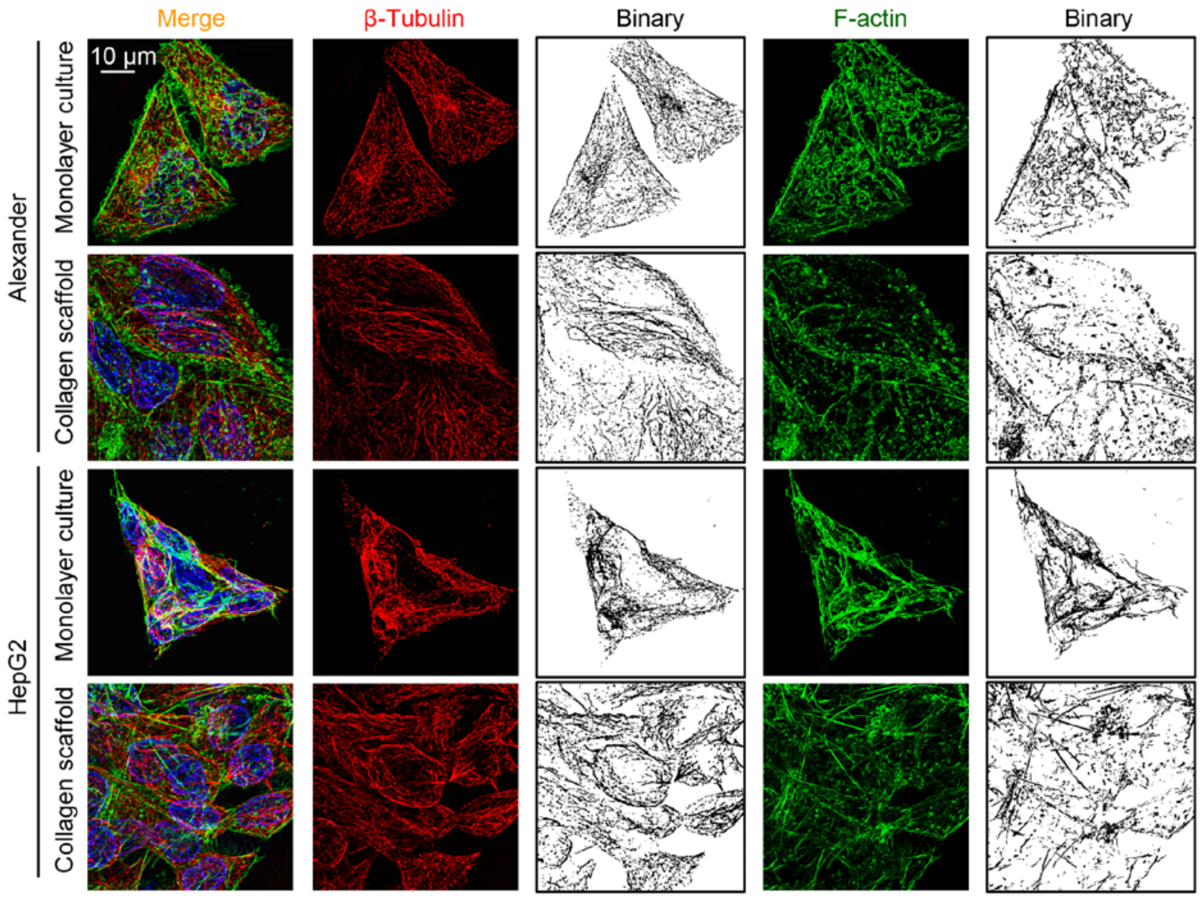

Figure S6. Maximum intensity projections of cytoskeleton remodeling under culturing in collagen scaffolds. Cells were grown either in standard monolayer culture or in collagen scaffolds, then fixed, permeabilized and stained for F-actin (green) and tubulin (red). Hoechst 33342 (blue) dye was used to counterstain nuclei. Labeled cells were then imaged by confocal microscopy, and the image was processed using ImageJ software (NIH). Binarization was done using ImageJ software (NIH).

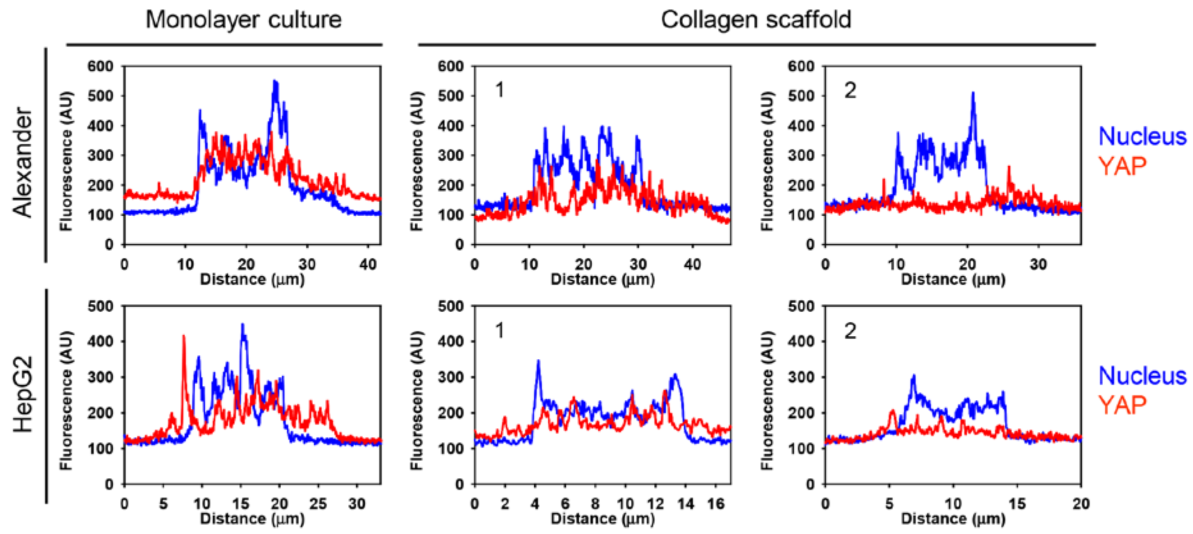

Figure S7. Sub-cellular localization of YAP under culturing in collagen scaffolds. Representative linescans of confocal microscopic images shown in Figure 4D.

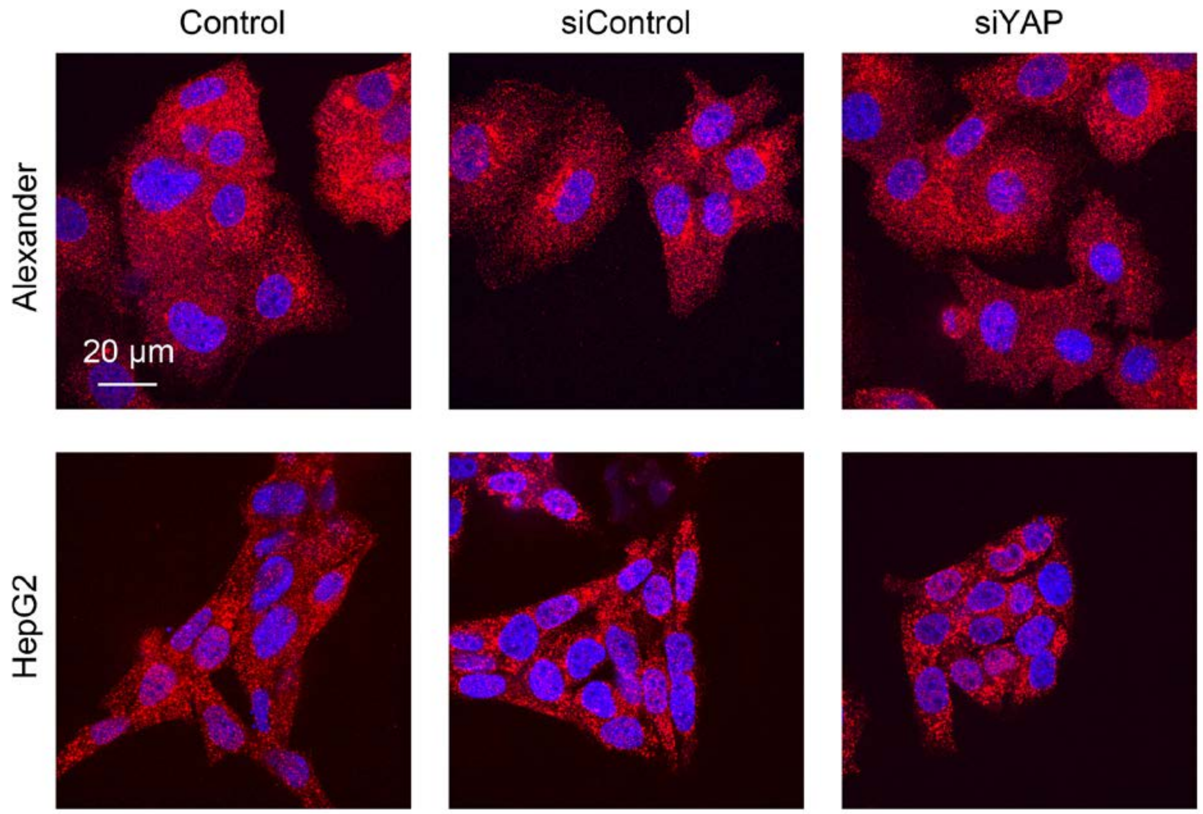

Figure S8. Sub-cellular distribution of pmTOR upon YAP siRNA transfection. Immunostaining analysis for pmTOR (red) in Alexander and HepG2 cells transfected with YAP siRNA for 48 h. Hoechst 33342 (blue) dye was used to counterstain nuclei. Labeled cells were then imaged by confocal microscopy, and the image was processed using ImageJ software (NIH). Representative confocal microscopic images.

Uncropped immunoblot scans

Figure 2F

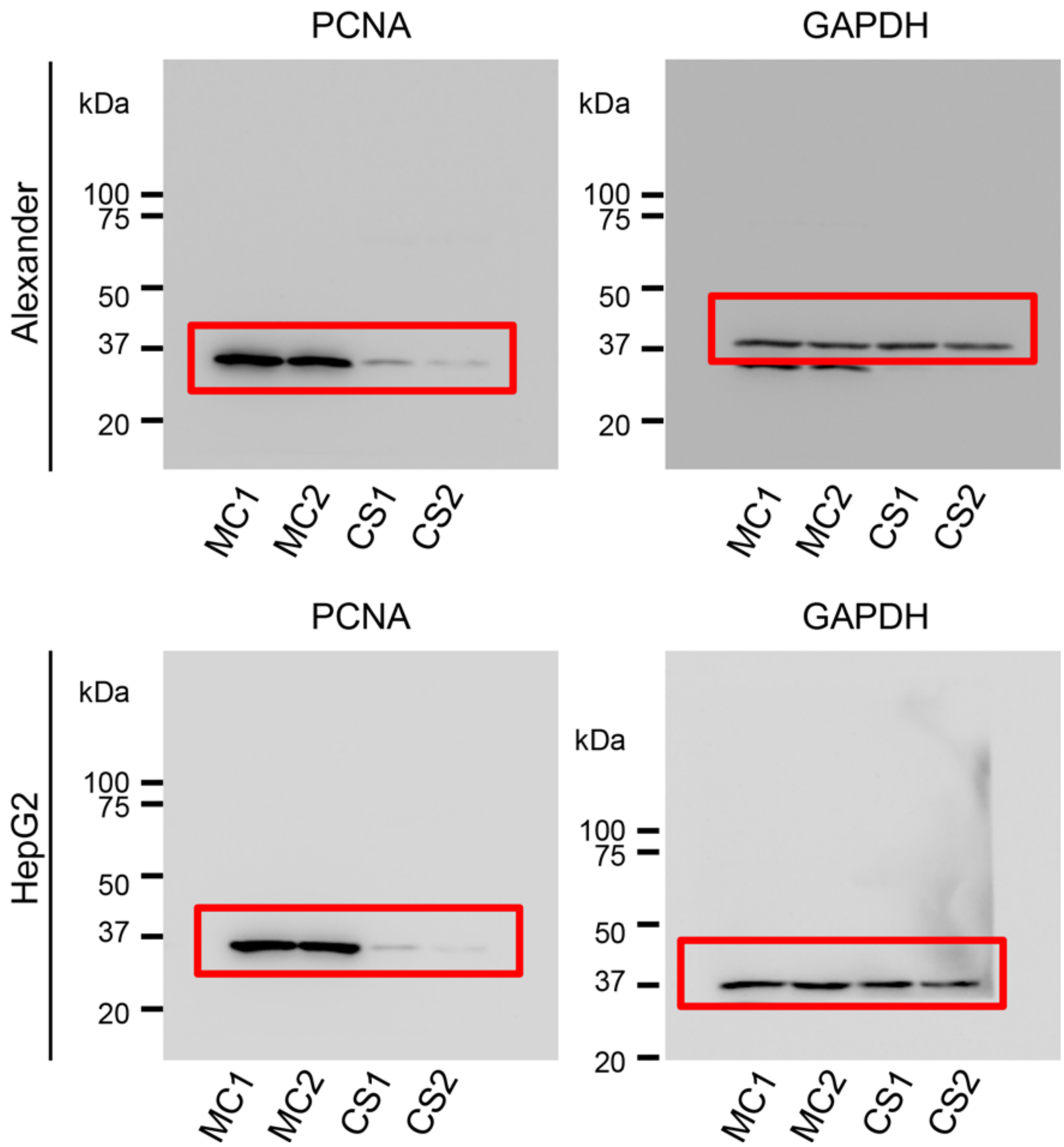

Figure 3F

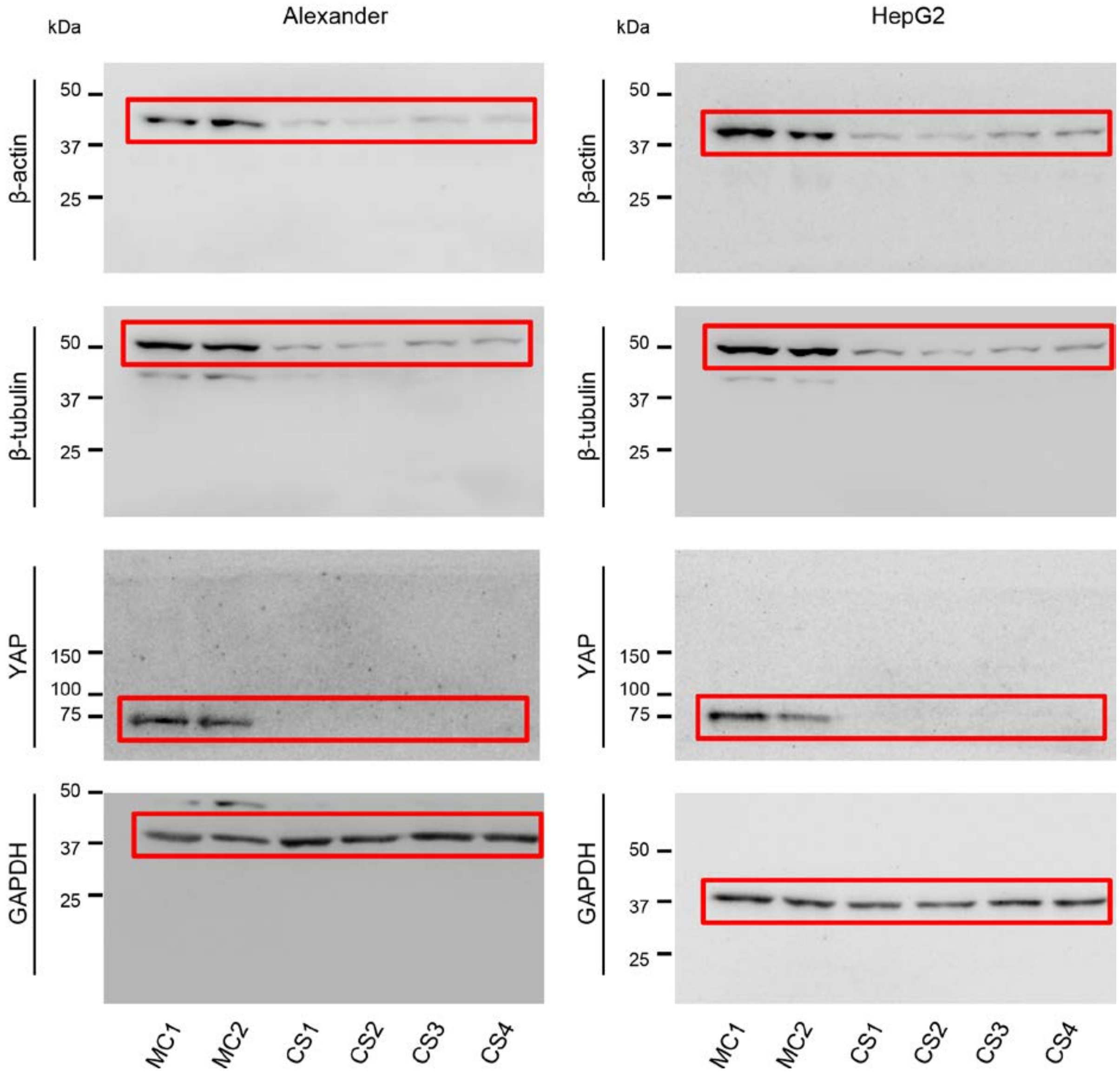

Alexander
HepG2
kDa
kDa
β-actin
β-tubulin
YAP
GAPDH
50
37
25
150
100
75
MC1
MC2
CS1
CS2
CS3
CS4

Replicates for Figure 3F

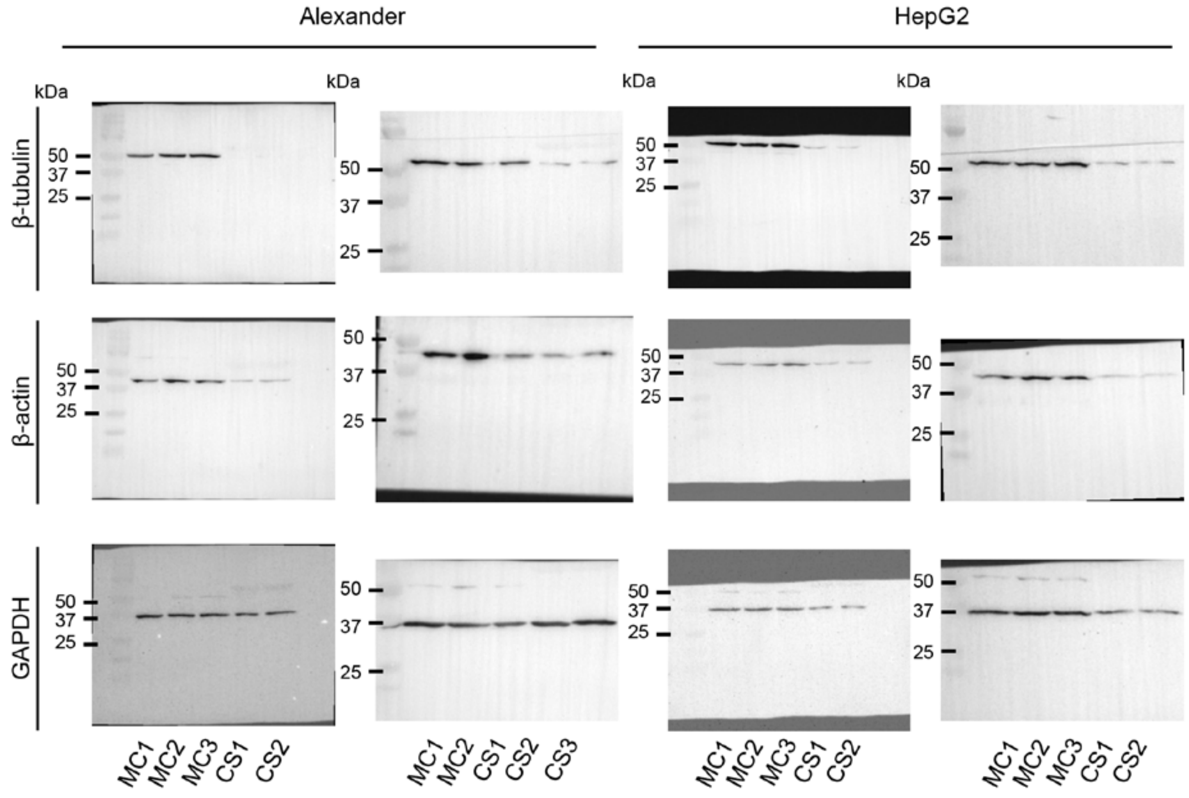

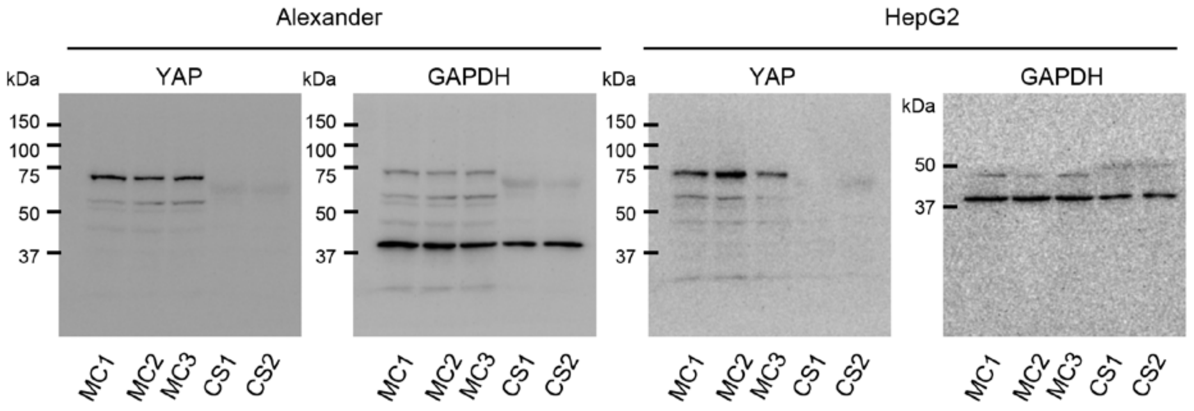

Figure 5A

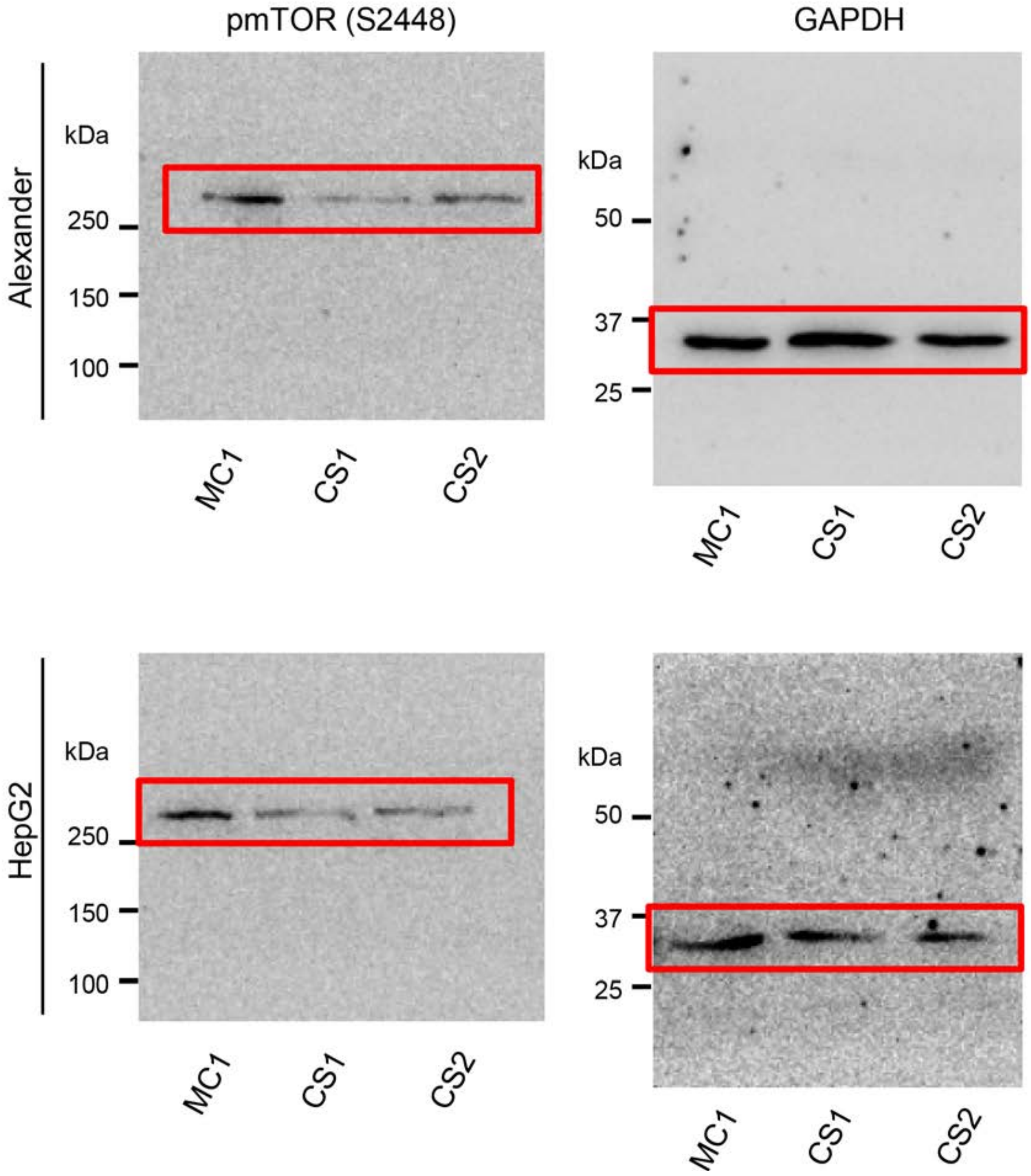
pmTOR (S2448)
GAPDH
Alexander
kDa
250
150
100
kDa
50
37
25
MC1
CS1
CS2
MC1
CS1
CS2
HepG2
kDa
250
150
100
kDa
50
37
25
MC1
CS1
CS2
MC1
CS1
CS2

Figure 6C

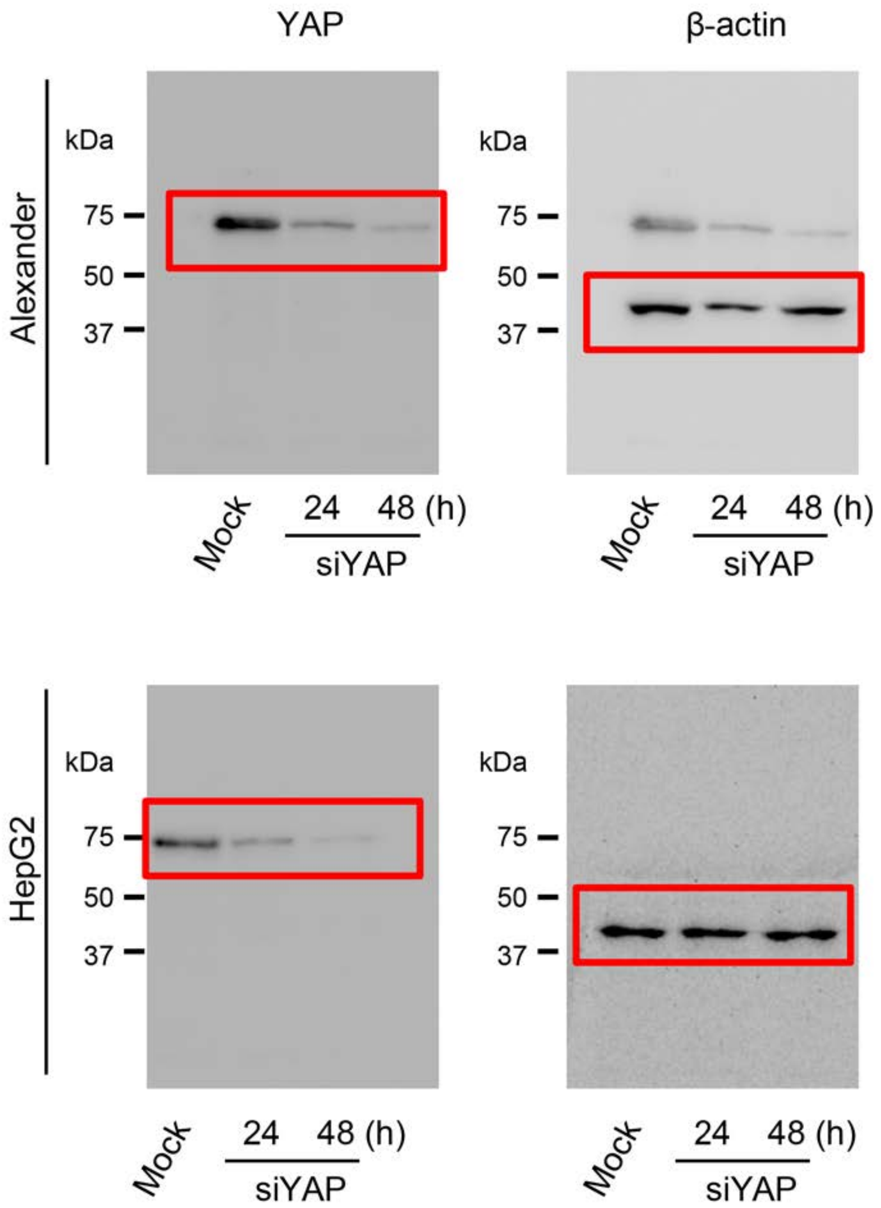

Figure 6E

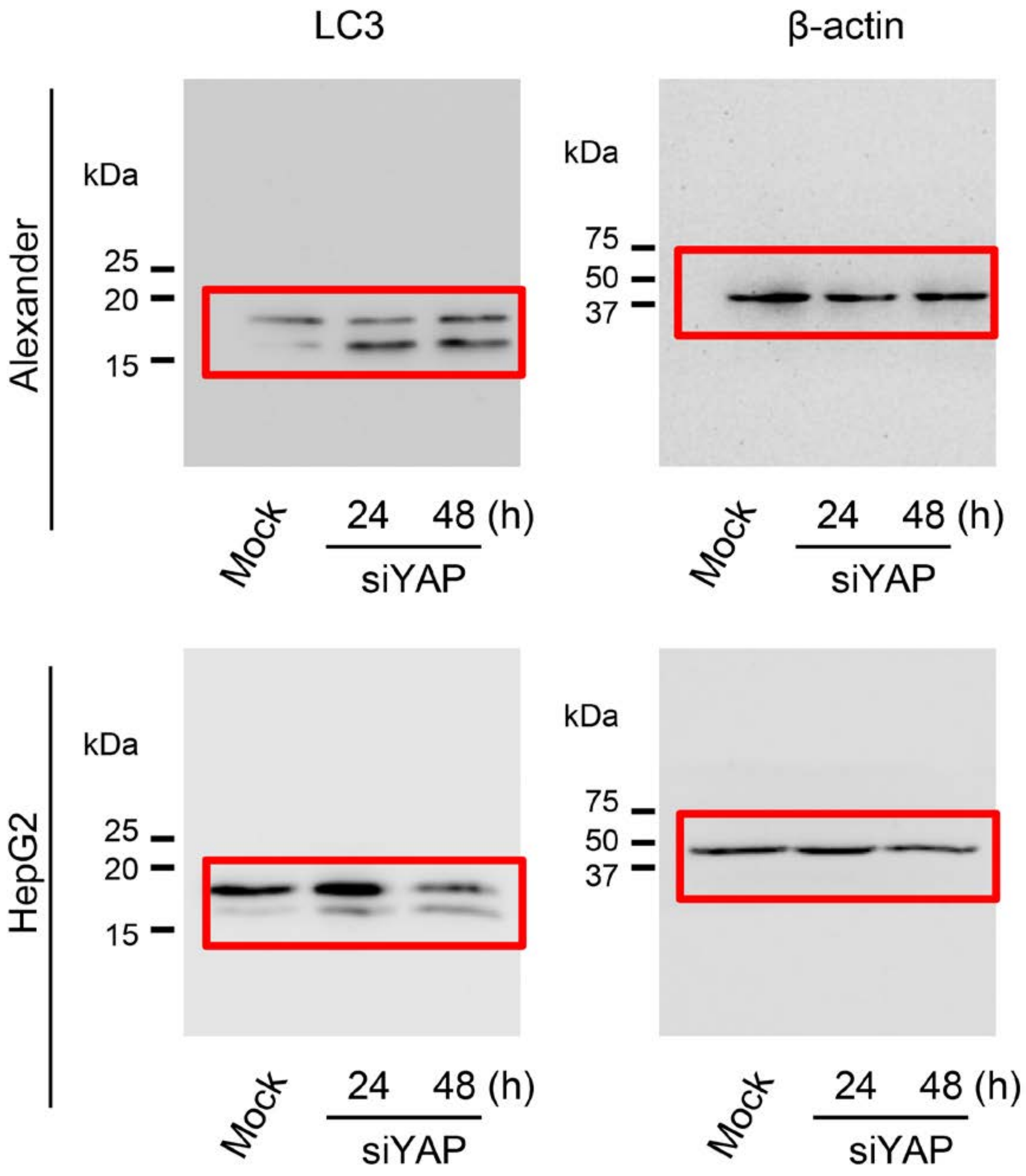
LC3
β-actin
Alexander
kDa
25
20
15
kDa
75
50
37
Mock
24
48 (h)
siYAP
Mock
24
48 (h)
siYAP
HepG2
kDa
25
20
15
kDa
75
50
37
Mock
24
48 (h)
siYAP
Mock
24
48 (h)
siYAP

Figure 7B

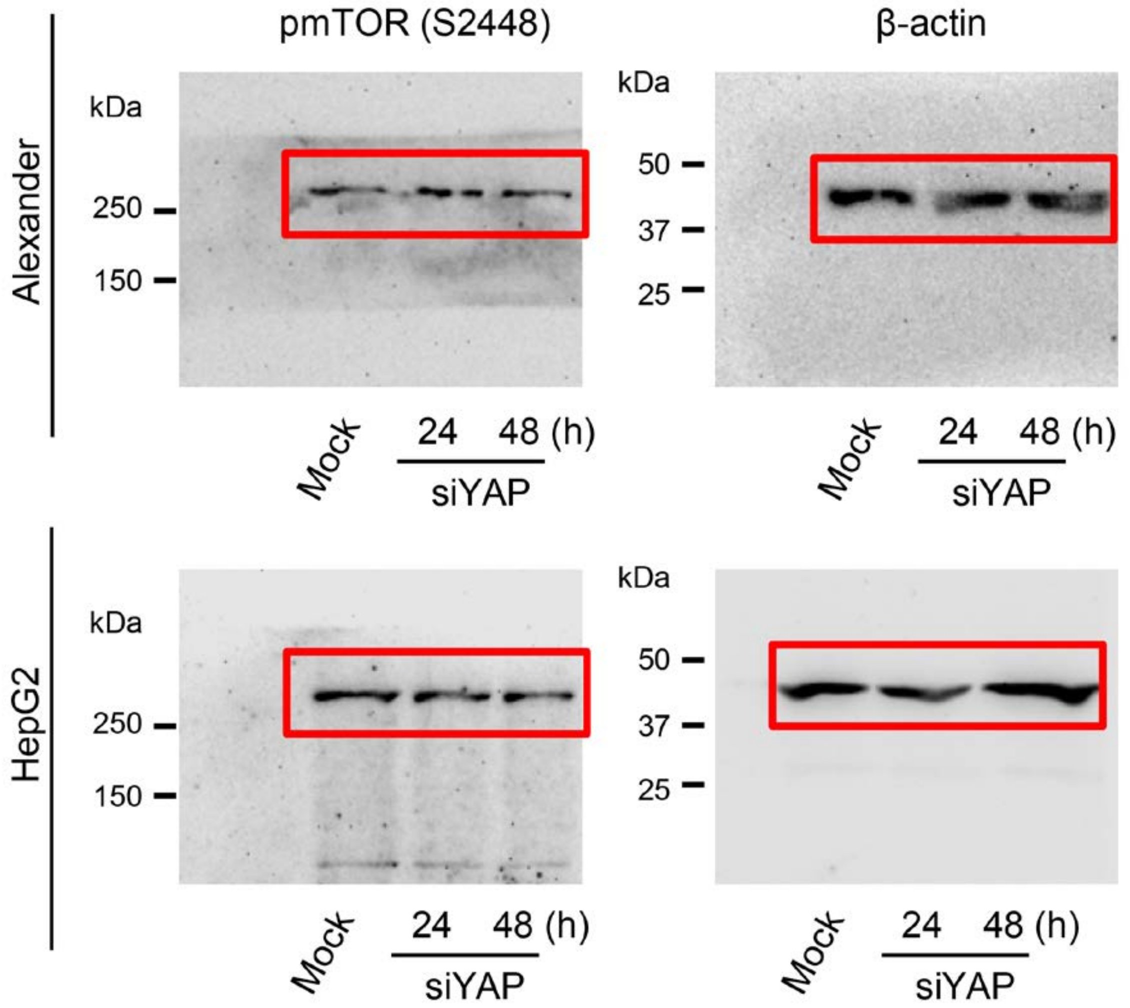
pmTOR (S2448)
β-actin
Alexander
kDa
250
150
50
37
25
Mock
24
48 (h)
siYAP
HepG2
